# Synovium microenvironment-responsive injectable hydrogel inducing modulation of macrophages and elimination of synovial fibroblasts for enhanced treatment of rheumatoid arthritis

**DOI:** 10.1186/s12951-024-02465-w

**Published:** 2024-04-17

**Authors:** Yiqun Wu, Yu Ge, Zhongshi Wang, Ying Zhu, Tianli Tian, Jun Wei, Yu Jin, Yi Zhao, Qiang jia, Jun Wu, Liang Ge

**Affiliations:** 1 State Key Laboratory of Natural Medicines, School of Pharmacy, China Pharmaceutical University, Nanjing, 210009 Jiangsu China; 2 Guangdong Provincial Key Laboratory of Malignant Tumor Epigenetics and Gene Regulation, Sun Yat-sen Memorial Hospital, State Key Laboratory of Oncology in South China, Guangzhou, 510120 China; 3 Bioscience and Biomedical Engineering Thrust, The Hong Kong University of Science and Technology (Guangzhou), Nansha, Guangzhou, 511458 China; 4 Division of Life Science, Hong Kong University of Science and Technology, Hong Kong SAR, 999077 China; 5 Department of Pharmacy, The Affiliated Hospital of Nantong University, Jiangsu, 226006 China; 6 Department of Pharmacy, The First Affiliated Hospital of Soochow University, Suzhou, 215026 Jiangsu China; 7 School of Biomedical Engineering, Shenzhen Campus of Sun Yat-sen University, Shenzhen, 518107 China; 8 Guangzhou City Polytechnic, Guangzhou, 510520 Guangdong China

**Keywords:** Rheumatoid arthritis, Bismuthene nanosheet, pH sensitive injectable hydrogel, Synovial fibroblasts, Modulation of macrophage

## Abstract

Rheumatoid arthritis (RA) is a progressive autoimmune disease accompanied by joint swelling, cartilage erosion and bone damage. Drug therapy for RA has been restricted due to poor therapeutic effect, recurrence and adverse effects. Macrophages and synovial fibroblasts both play important roles in the pathology of RA. Macrophages secrete large amount of pro-inflammatory cytokines, while synovial fibroblasts are tightly correlated with hypoxia synovium microenvironment, cytokine release, recruitment of pro-inflammatory cells, bone and cartilage erosion. Therefore, in this timely research, an injectable and pH-sensitive peptide hydrogel loading methotrexate (MTX) and bismuthene nanosheet/polyethyleneimine (BiNS/PEI) has been developed to reduce the activity of macrophages and eliminate over-proliferated synovial fibroblasts simultaneously. MTX can reduce the cytokine secretion of macrophages/anti-apoptosis property of synovial fibroblasts and BiNS/PEI can eliminate synovial fibroblasts via photodynamic therapy (PDT) and photothermal therapy (PTT) routes. The hydrogel was injected into the acidic inflammatory synovium for precise targeting and served as a drug reservoir for pH responsive and sustained drug release, while improving the bioavailability and reducing the toxicity of MTX. Excellent therapeutic efficacy has been achieved in both in vivo and in vitro studies, and this unique drug delivery system provides a new and robust strategy to eliminate synovial fibroblasts and modulate immune system for RA treatment in clinical.

**Graphic Abstract:**

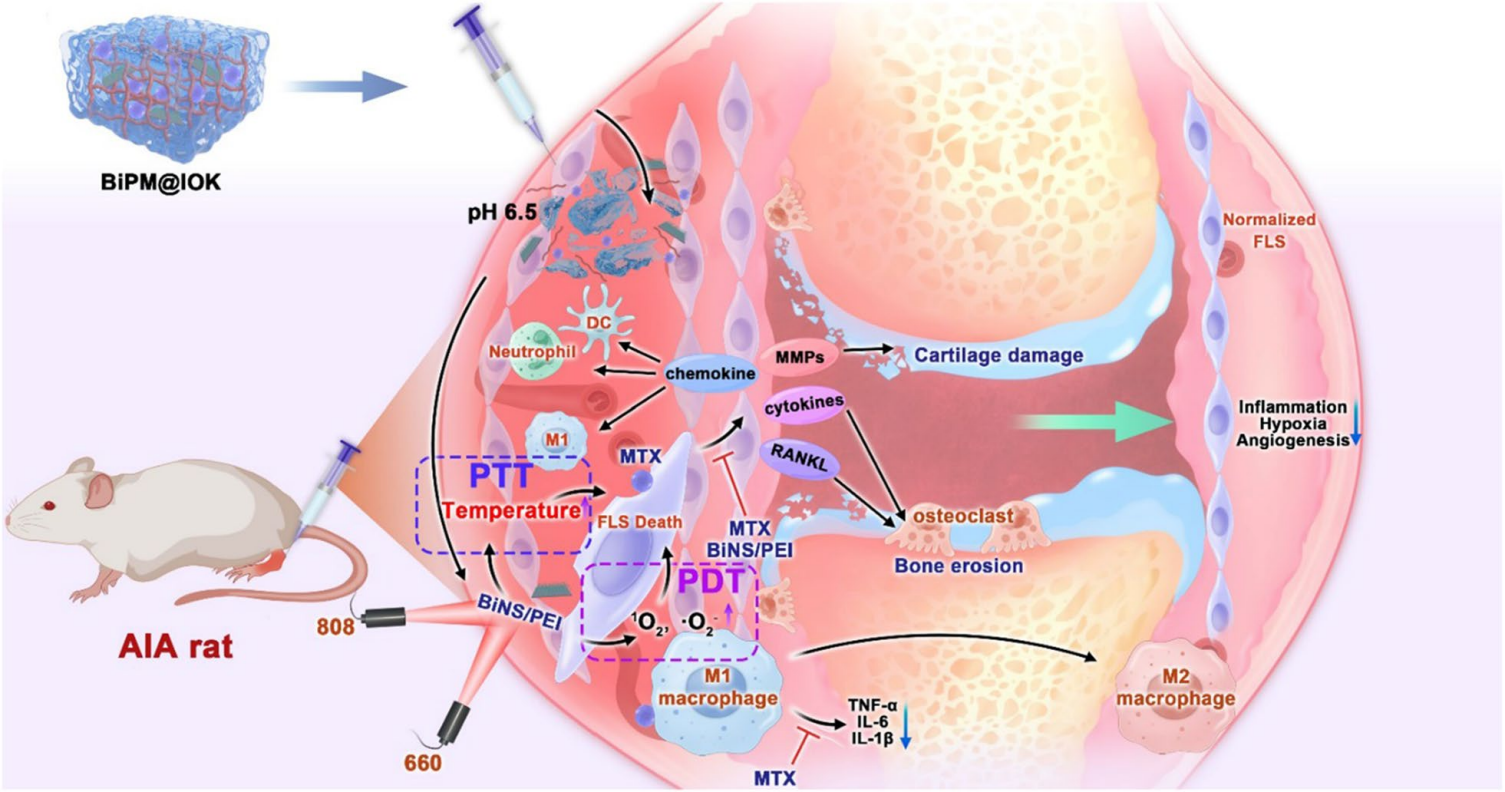

**Supplementary Information:**

The online version contains supplementary material available at 10.1186/s12951-024-02465-w.

## Introduction

Rheumatoid arthritis (RA) is a progressive autoimmune disease caused by gene, environment, immune factors and affects about 1% of the population worldwide [1]. RA is characterized by joint swelling, pain, cartilage-bone damage and lead to systemic abnormal immune responses. With the prolongation of the disease course, the incidence of disability and functional limitation increases significantly [2, 3]. Meanwhile, the risk of severe infection, respiratory disease, cardiovascular disease, osteoporosis and cancer accompanied by RA seriously impact on patient's quality of live [4, 5].

Synovitis is the main symptom of RA. Synovium is a thin membrane between fibrous joint capsule and synovial cavity. The intimal lining of synovium is close to synovial cavity and its outer layer is loosely connected with sub-lining. Synovial fibroblasts (FLS) in the intimal lining maintain the stability of extracellular matrix by secreting matrix components (such as collagen, laminin, etc.) and enzymes which degrade extracellular matrix (such as matrix metalloproteinase, cathepsin, etc.), maintaining the dynamic stability of the extracellular matrix. Besides, FLS also secrete hyaluronic acid and lubricin which play a significant role in lubrication, nutrition and protection of articular cartilage [6]. Macrophages, synovial fibroblasts, adipocytes, blood vessels and lymphatic vessels were sparsely distributed in the synovium sub-lining [7]. In the pathological model of RA, pro-inflammatory cells such as macrophages, T cells, dendritic cells, neutrophils massively infiltrate the synovium tissue and FLS rapidly proliferate under the stimulation of pro-inflammatory cytokines [5]. Among them, macrophages show a M1 pro-inflammatory phenotype and play a pivotal role in the secretion of pro-inflammatory cytokines such as tumor necrosis factor-α (TNF-α), interleukin-1β (IL-1β) and interleukin-6 (IL-6) to promote the development of inflammation. Therefore, inhibiting the inflammatory factors secreted by macrophages is extremely worthwhile [1, 8]. On the other hand, FLS play a central role in local immunity, inflammation maintenance, cartilage and bone damage in RA. FLS can be divided into THY^+^ FLS and THY^−^ FLS according to whether THY (also known as CD90) is expressed on the surface. THY^−^ FLS cells located in the intimal lining secrete matrix metalloproteinase (MMP) such as MMP3, MMP9 and MMP13, leading to cartilage damage [9, 10]. THY^+^ FLS in the sub-lining secretes proinflammatory cytokines such as IL-6, prostaglandins, leukotrienes and chemokines such as CCL2, CCL5, CCL12, which promoted the activation and attraction of immune cells. At the same time, it secretes receptor activator for nuclear factor-κB ligand (RANKL), activating osteoclasts, leading to bone erosion and inhibiting natural bone repair [11–13]. Besides, the over-proliferation of FLS in and the infiltration of immune cells create hypoxia microenvironment, inducing the polarization of macrophages from anti-inflammatory M2 type to pro-inflammatory M1 type [14]. Meanwhile, the elevated expression of hypoxia-inducible factor HIF-1α promotes angiogenesis, thereby enhancing the infiltration of immune cells into the synovium [12, 13, 15]. In conclusion, it is crucial to eliminate the proliferated FLS and inhibit the secretion of inflammatory cytokines by macrophages to cure RA.

At present, the most important clinical treatment method for RA is drug therapy. The commonly used drugs include the following four categories: (1) Traditional disease modifying antirheumatic drugs (DMARDs): include Methotrexate (MTX), leflunomide, sulfasalazine, etc. (2) Biological DMARDs: Therapeutic targets of DMARDs are specific cytokines that cause the disorder of immune response, such as anti-TNF agent infliximab, IL-6 receptor inhibitor tocilizumab, etc. (3) Cell-targeted biological DMARDs, including anti-CD20 antibody rituximab, T cell co-stimulation inhibitor abatacept, etc. (4) Glucocorticoids and non-steroidal anti-inflammatory drugs. Among them, methotrexate has been widely used in the clinical treatment of RA [16]. As a dihydrofolate reductase inhibitor, the mechanism of MTX in the treatment of RA is not fully understood and adenosine pathway is considered to be the main anti-inflammatory mechanism [17]. At the same time, whether it is orally or parenteral administrated, the half-life of MTX is extremely short. The value is only about 6 h, and it cannot be detected in the serum 18 h after administration [17]. Therefore, elevating the targeting ability of MTX to reduce systemic toxicity, enhancing the sustained release ability of MTX to reduce the frequency of administration and improving the compliance of administration are significant for its treatment efficacy.

MTX can inhibit the activity of macrophages and reduce the secretion of inflammatory factors. However, there are very limited methods to eliminate the excessive proliferation of synovial fibroblasts in the pathological environment of RA except synovectomy [18]. RA is known as an “immortal cancer” and FLS have invasive and anti-apoptotic properties similar to tumor cells [9, 10]. Therefore, we speculated that therapy methods of tumor also have the potential to relieve the symptoms of RA. We turned our attention to two-dimensional nanomaterial, a new class of ultra-thin layered materials with high anisotropy and planar structure, and their longitudinal structure is single or multiple atomic layers [19, 20]. They have attracted widespread attention in energy, physics, biomedicine and other fields due to their excellent physical and chemical properties [21–24]. Among them, two-dimensional single-element nanomaterials (Xenes) are mainly composed of elements of group IIIA, IVA and VA, including borene, silanene, phosphoene, arsenene, antimonene, bismuthene and Te, etc. [21]. In terms of performance, Xenes have a tunable band gap and could realize the interaction with electromagnetic waves from the ultraviolet to the near-infrared spectral region, showing great application potential [25]. Bismuth (Bi) is a group VA diamagnetic heavy metal element. Unlike group IIIA and IVA elements, Xenes composed of group VA elements such as bismuthene usually form similar honeycomb-like monolayer fold structures with electron rich surface [26]. Two-dimensional bismuth nanosheet (BiNS) has a lattice structure similar to graphene and strong spin–orbit coupling so that it has great potential as topological semi-metals or semiconductors. With the size of bismuth reduced to the nanoscale, bismuth is converted from a metal to a semiconductor material according to quantum confinement effects to produce a unique photoelectric effect [27–29]. In this research, we aimed to develop the photodynamic properties of BiNS in combination with their photothermal properties to eliminate over proliferated FLS. To improve the cellular uptake ability of BiNS for photodynamic properties and stability in water, we introduced the cationic material polyethyleneimine with molecular weight of 10 k and BiNS/PEI nanoparticles were formed by electrostatic adsorption.

**Scheme 1:**
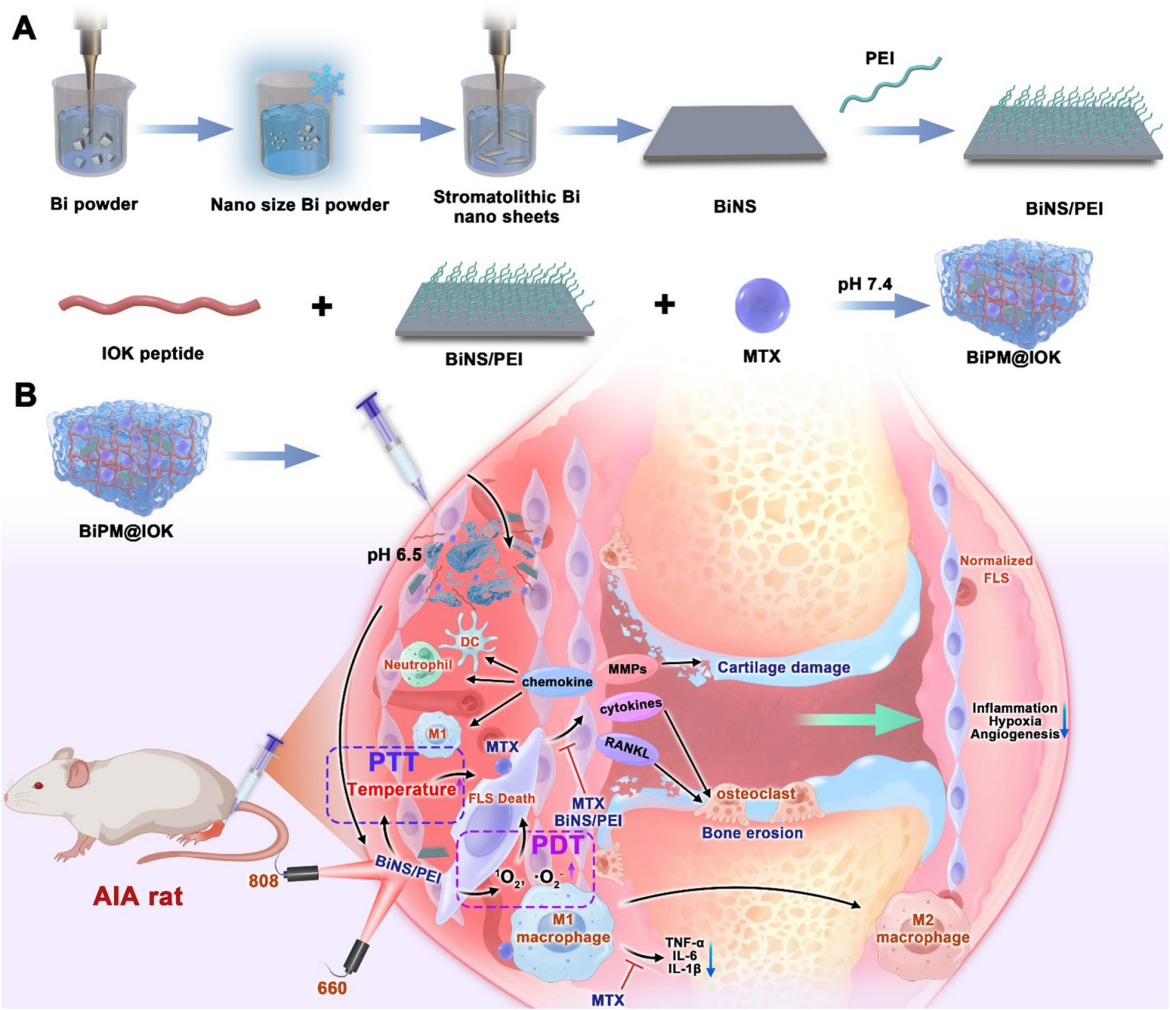
**A** Preparation of BiNS, BiNS/PEI and BiPM@IOK. **B** Systematic treatment strategies of BiPM@IOK: Combining modulating immune system and eliminating over-proliferated synovial fibroblasts together for enhanced RA treatment

Consistent with MTX, BiNS/PEI was aimed to target inflammatory synovium tissues. However, neither BiNS nor MTX had the active targeting ability. To improve the targeting ability and sustained release property for reducing the number of drug administration, we designed a hydrogel carrier to encapsulate BiNS/PEI and MTX for *in-situ* synovium injection. Hydrogel is a three-dimensional network structure material formed by physical or chemical cross-linking of hydrophilic materials [30–32]. Due to its high-water content, low invasiveness and adjustable mechanical properties, hydrogel has become an excellent biological material [33–35]. Hydrogel formed by self-assembly of polypeptide is called peptide hydrogel, which has advantages such as low immunogenicity, sequence diversity, good functionality, easy modification, large-scale synthesis, inherent biocompatibility and biodegradability over traditional polymer hydrogel [36, 37]. In this study, we designed a new pH sensitive IOK peptide (Sequence: IVNFOFLSK) hydrogel for RA synovium implantation of BiNS/PEI and MTX. (1) The hydrophobic amino acid isoleucine (Ile, I) was used to enhance the mechanical strength and stability, (2) Amino acid ornithine (Orn, O, pI = 10.80) at position 5 and lysine (Lys, K, pI = 9.74) at position 9 make IOK easier to be protonated under acid conditions to improve the pH sensitivity of the peptide. BiNS/PEI and MTX can be encapsulated by IOK peptide under physiological pH condition and self-assembled into BiPM@IOK hydrogel. IOK hydrogel was able to function as a drug reservoir to enhance the ability of sustained drug release and reduce the number of drug administration to improve compliance. When the hydrogel was injected into the synovium of the inflamed joint in situ, the degree of protonation of IOK peptide increased under the stimulation of the slightly acidic synovium microenvironment [38, 39] so that the electrostatic repulsion enhanced, leading to the collapse of hydrogel and accelerated released of BiNS/PEI and MTX directly in synovium. Under inflammatory conditions, folate receptor was highly expressed on surface of macrophages which was convenient for cell uptake [40]. Research showed that cationic nanoparticles were conducive for tissue retention [41–45] so that released BiNS/PEI with positive charge can residue in synovium for a long period which was beneficial for cell uptake. Furthermore, another research reported that peptides with hydrophobic sequence can enhance the targeting and residence properties of cationic nanoparticles in joint tissue [46]. Due to the presence of Ile, Val and Leu, IOK peptide had the potential to provide a certain degree of hydrophobicity which was beneficial for enhancing the synovium targeting and retention of BiNS/PEI. Photothermal and photodynamic performances of BiNS/PEI eliminated over proliferated FLS and relieved synovium microenvironment. MTX inhibited the secretion of inflammatory cytokines by macrophages in coordination with BiNS/PEI to treat RA (Scheme 1).

## Results and discussion

### Preparation and characterization of BiNS and BiNS/PEI

**Fig. 1:**
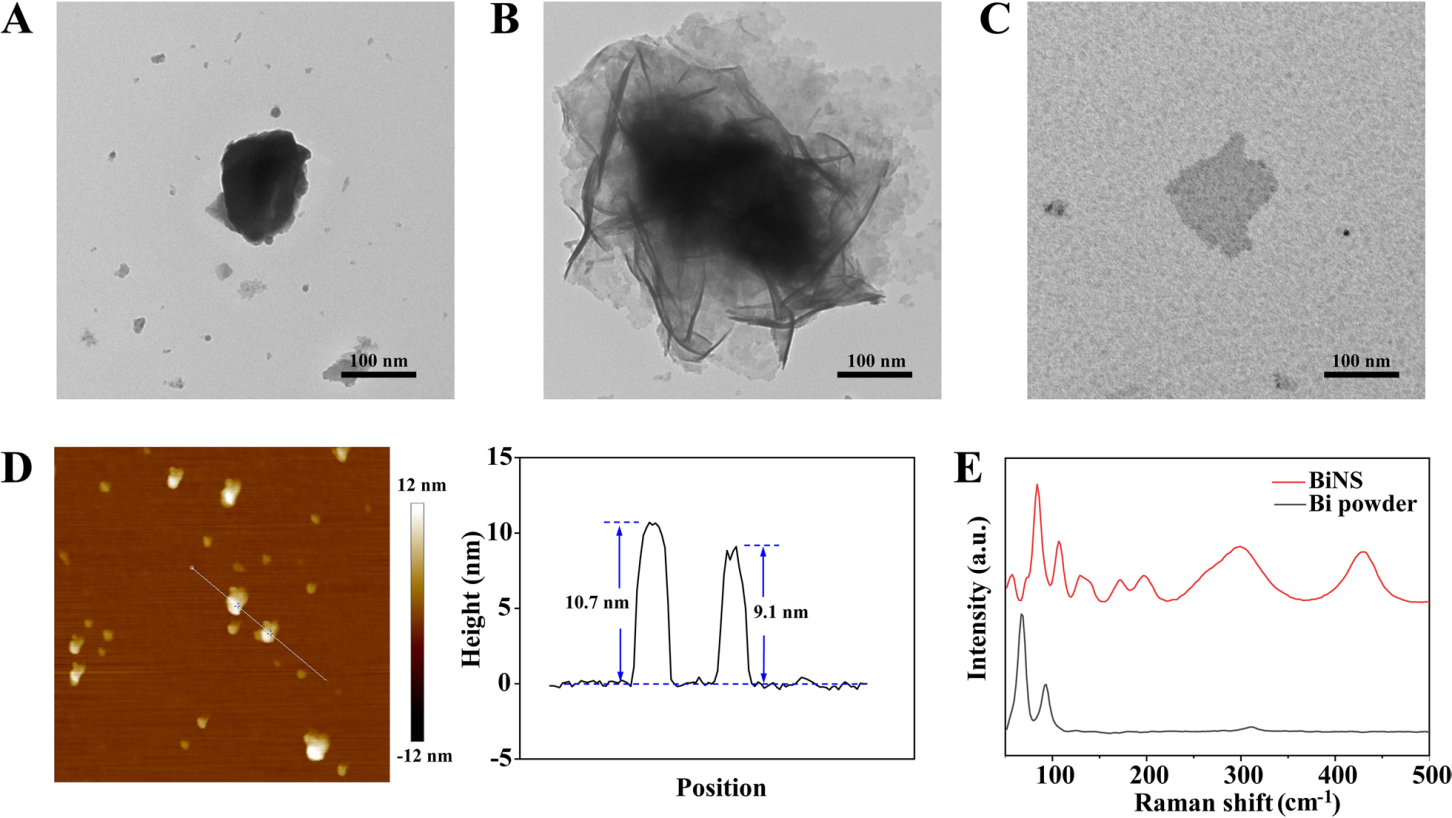
Characterization of BiNS. TEM images of Bi powder with different treatments, including probe ultrasonication for the first time (**A**), freezing thawing (**B**) and probe ultrasonication for the second time (**C**). Scale bar: 100 nm. **D** AFM image of BiNS and height profiles along the white lines in the image. **E** Raman spectra of Bi powder and BiNS

**Fig. 2:**
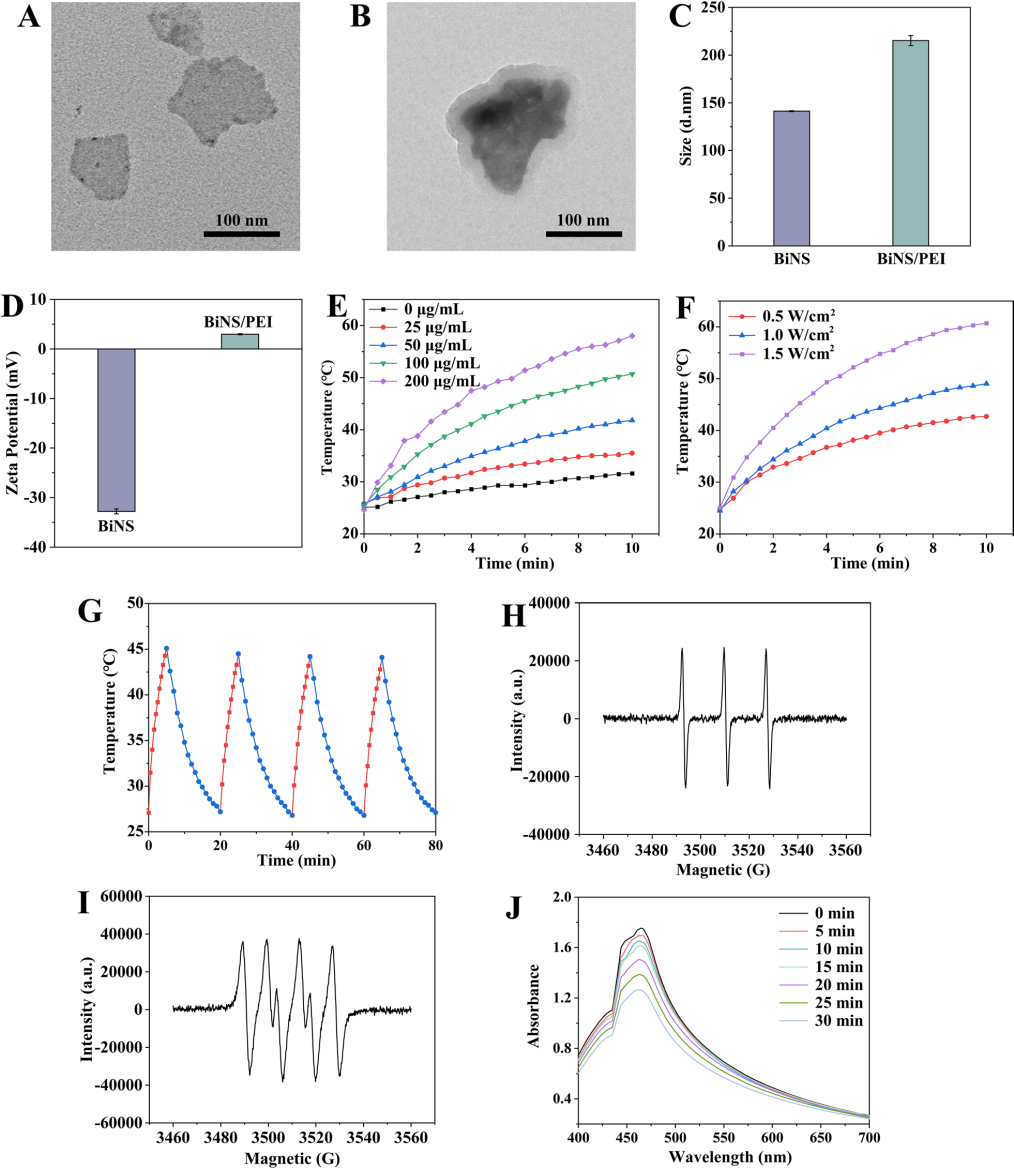
Characterization of BiNS/PEI. TEM images of BiNS (**A**) and BiNS/PEI (**B**). Scale bars were 100 nm. Size (**C**) and Zeta potential (**D**) of BiNS and BiNS/PEI. **E** Temperature change of BiNS/PEI with different concentrations under NIR (808 nm, 1 W/cm^2^) irradiation. **F** Temperature increases of 100 μg/mL BiNS/PEI under NIR (808 nm) laser irradiation with the power of 0.1, 1, 1.5 W/cm^2^. **G** Temperature circulation curves of 100 μg/mL BiNS/PEI under NIR (808 nm, 1 W/cm^2^) irradiation. ^1^O_2_ (H) and O_2_^−^ (**I**) generation of BiNS/PEI characterized by ESR spectra under 0.1 W/cm^2^ 660 nm laser irradiation (**J**) UV–vis absorption spectra of DPBF in 100 μg/mL BiNS/PEI suspension under 0.1 W/cm^2^ 660 nm laser irradiation for different times

BiNS was prepared by liquid phase stripping in anhydrous ethanol through “ultrasonication-freezing-ultrasonication”. The changes in material morphology during the process were observed by TEM. After the initial ultrasonication for 12 h, the transverse size of Bi powder was reduced to 100–200 nm, but it still showed a certain thickness of lumps (Fig. 1A). After freezing and thawing treatment, the massive structure of Bi was destroyed as it showed a slightly dispersed layered structure, and the surrounding of the massive Bi appeared a thin sheet shape (Fig. 1B). Since Bi is a metal with natural layered structure [47], water molecules can be inserted freely between the bismuth layers. When frozen at -80℃, the volume expansion of water between the layers can counteract the van der Waals force and destroy the tight layer structure of bismuth. After that, another ultrasonication for 12 h was performed and uniform ultrathin Bi nanosheets (BiNS) with a transverse size of about 140 nm can be easily obtained (Fig. 1C). Using anhydrous ethanol as the liquid phase can prevent the rapid oxidation of ultrathin Bi nanosheets during ultrasonication. AFM was used to measure the thickness of BiNS obtained by the liquid phase stripping method. As shown in Fig. 1D, the longitudinal dimensions of BiNS were all about 10 nm, indicating that BiNS with thin layer structure could be obtained by this method. The ultrathin two-dimensional structure improved the specific surface area and reactivity of Bi. It is endowed with unique optical, electrical and catalytic properties. The structure changes of Bi powder and BiNS before and after stripping were investigated by Raman spectroscopy. As shown in Fig. 1E, the Raman characteristic peaks of Bi powder at 68.7 and 91.4 cm^−1^ corresponded to in-plane Eg and out-of-plane A1g vibration modes [27, 48], respectively. Raman spectra of BiNS showed that the Raman characteristic peaks at 83.8 cm^−1^ and 106.5 cm^−1^ were similar with those in the Raman spectra of Bi powder. However, there was a slight blue shift in the characteristic peaks which might be caused by the decrease in the number of material layers when BiNS was stripped from powder to nanosheet. This phenomenon had also been observed in previous studies of Bi nanosheets and other two-dimensional nanomaterials [27, 28]. The other five Raman peaks at 129.1, 172.2, 196.5, 298.2, 429.2 cm^−1^ were the Raman peaks of Bi_2_O_3_ [48]. The structure of Bi powder and two-dimensional BiNS was further characterized by X-ray diffractometer. Except for the presence of diffraction peaks near 33°, the diffraction peaks of Bi powder and BiNS were consistent with the patterns of standard Bi element (PDF#85–1329) (Additional file 1: Fig. S1), verifying that the composition of BiNS obtained did not change. The diffraction peaks around 33° in the XRD pattern of Bi powder and BiNS also appear in the standard pattern of Bi oxide (PDF#71–2274), indicating that the process of oxidation was inevitable during storage, preparation and detection. Finally, TEM and EDS were combined to conduct element analysis of the prepared BiNS. As shown in Additional file 1: Fig. S2, Bi and oxygen elements distributed on the surface of the nanosheet. The results showed that the prepared BiNS only contained Bi and O elements and no other impurities were doped in it. Due to its high reactivity, BiNS was inevitably oxidized during preparation and storage. The successfully prepared BiNS could be uniformly dispersed in anhydrous ethanol (Additional file 1: Fig. S4A). However, the lyophilized BiNS were difficult to re-disperse in water (Additional file 1: Fig. S4B) and ultrasonic dispersion caused the BiNS to be oxidized instantly (Additional file 1: Fig. S4C). For improving the stability of the BiNS in water and enhance the ability of cell uptake, cationic polymer polyvinyl imide (PEI 10 k) and BiNS were used to construct nanoparticle BiNS/PEI through electrostatic adsorption. After preparation, BiNS/PEI were ultrasonically suspended in deionized water. The status of sample was stable. No precipitation or oxidation whitening was observed (Additional file 1: Fig. S4D). After BiNS/PEI deionized water suspension was stored in a refrigerator at 4 ℃ for 3 months, the sample remained relatively evenly dispersed in deionized water and the morphology was consistent with that of the newly prepared samples (Additional file 1: Fig. S4E). TEM was used to characterize the micro morphologies of BiNS before and after modification of PEI, as shown in Fig. 2A, B, compared to bare BiNS, the transverse size of BiNS/PEI obviously increased. PEI was uniformly adsorbed on the surface of BiNS by electrostatic force to form a coating layer. DLS was used to test the particle size of BiNS and BiNS/PEI. As shown in Fig. 2C, the average particle size of BiNS was 141.23 nm and the average particle size increased to 215.17 nm after PEI modification. Subsequently, we discussed the particle stability of BiNS/PEI at 37 ℃, 25 ℃ and 4 ℃ within 7 days. As shown in Additional file 1: Fig. S3, particle size of BiNS/PEI gradually increased in this period. Meanwhile, with the elevation of surrounding temperature, the increase in particle size became larger. Delightfully, the increasing rates under three temperatures were all relatively low, indicating the excellent stability of BiNS/PEI. Average Zeta potential of BiNS is − 32.77 mV. After 10 kDa PEI modification, the value changed from negative to positive, reaching 2.95 mV, which also proved the successful preparation of BiNS/PEI. The slight positive charge on the surface of 2D Bi nanosheets can ensure the biosafety, promote cell uptake of negative charge BiNS to exert the photodynamic function and enhance its synovium retention [41–45].

### In vitro photothermal performance of BiNS/PEI

Then we evaluated the in vitro photothermal properties of BiNS/PEI as an agent for photothermal therapy. Firstly, the photothermal properties of BiNS/PEI with different concentrations were studied to select the appropriate concentration for subsequent experiments. As shown in Fig. 2E, when the concentration of BiNS/PEI was 0 μg/mL, the water temperature only slightly increased under the irradiation of 1W/cm^2^ 808 nm laser, while the temperature of all the other concentrations of BiNS/PEI suspension significantly increased and the change was concentration dependent. When the concentrations were 25 μg/mL, 50 μg/mL, 100 μg/mL and 200 μg/mL, the temperature of BiNS/PEI suspension within 5 min increased by 6.9 ℃, 10.7 ℃, 18.2 ℃ and 24.7 ℃, respectively, compared with the initial temperature. Within 10 min, the temperature increased by 9.7 ℃, 16.1 ℃, 25.4 ℃ and 33.4 ℃, respectively. The results showed that BiNS/PEI can effectively convert near-infrared light into heat energy and the temperature for cell ablation can be reached after near-infrared light irradiation in a short time. Subsequently, we studied the photothermal properties of BiNS/PEI under the irradiation with different powers to select the appropriate irradiation power for subsequent experiments. As shown in Fig. 2F, the temperature increase of 100 μg/mL BiNS/PEI suspension had an obvious irradiation power dependence. When the laser power was 0.5 W/cm^2^, 1 W/cm^2^ and 1.5 W/cm^2^, the temperature of the suspension within 5 min increased by 13.3 ℃, 18.1 ℃ and 27.1 ℃, respectively, compared with the initial temperature. Within 10 min, the temperature increased by 17.9 ℃, 24.5 ℃ and 35.6 ℃, respectively. For balancing the effect of photothermal treatment and the skin tolerance temperature, we finally selected 100 μg/mL BiNS/PEI, 1 W/cm^2^ near infrared light irradiation power and 5 min irradiation time for follow-up experiments. Finally, the photothermal stability of 100 μg/mL BiNS/PEI suspension under 1 W/cm^2^ power of 808 nm near infrared light was verified by switching cycle experiments. As shown in Fig. 2G, a period of a single cycle was 20 min, including the temperature rise process of BiNS/PEI suspension irradiated for 5 min and the cooling process of natural cooling to the initial temperature for 15 min. In the four cycles, the temperature of BiNS/PEI suspension increased by 18 ℃, 17.3 ℃, 17.4 ℃ and 17.3 ℃ respectively after irradiation for 5 min. All the four temperature changing curves maintained similar warming and cooling trends. The results showed that BiNS/PEI had good photothermal stability which was benefit for flexible clinical treatments.

### In vitro photodynamic properties of BiNS/PEI

Electron spin resonance spectroscopy further validated the ability of BiNS/PEI to generate reactive oxygen species irradiated by red light with 660 nm wavelength. As shown in Fig. 2H, I, TEMP captured singlet oxygen (^1^O_2_) generated by BiNS/PEI in the system and displayed characteristic 1:1:1 ESR signal with three equal peaks. DMPO captured superoxide anion (O_2_^−^) produced by BiNS/PEI in the methanol system, displaying the ESR signal of characteristic 2:2:1:2:1:2 peaks. The results showed that BiNS/PEI can be used as photosensitizer to produce ROS under 0.1 W/cm^2^ 660 nm irradiation, thus playing a role in removing synovial fibroblasts. DPBF probe is a probe molecule that detects the production of reactive oxygen species by measuring its absorbance attenuation at the absorption peak and it was used to study the ability of BiNS/PEI to produce reactive oxygen species dominated by singlet oxygen under red light irradiation at 660 nm wavelength with 0.1 W/cm^2^ power. As shown in Fig. 2J, with the increase of the irradiation time, the absorbance of DPBF gradually decreased, indicating that BiNS/PEI had significant photodynamic properties.

### Preparation and characterization of BiPM@IOK hydrogel

The purity analysis of IOK polypeptide synthesized by solid-phase polypeptide synthesis was performed by High Performance Liquid Chromatography (HPLC). As shown in Additional file 1: Fig. S7, HPLC and peak data showed that the purity of the synthesized IOK polypeptide was 99.87%, meeting the purity requirements of the experiment. We used mass spectrometry to identify the structure of the synthesized IOK polypeptide. The molecular weight of IOK polypeptide was 1081.26 so that the theoretical value of [M + H]^+^ peak was 1082.26, the theoretical value of [M + 2H]^2+^ peak was 541.63. The mass spectrometry showed that the measured value of [M + H]^+^ peak of the synthesized polypeptide is 1081.19, and the measured value of [M + 2H]^2+^ peak was 541.83. All of them were consistent with the theoretical values (Additional file 1: Fig. S8) which proved that the synthesized peptide was the target IOK polypeptide. The initial pH value of IOK polypeptide solution was about 5.5. Under this acidic condition, the polypeptide presented a clear solution state. The polypeptide solution gradually became cloudy and viscous with the increase of pH value and the fluidity became weaker. When the pH value reached 7.0, the polypeptide solution presented an unstable gel state. After the neutral condition of pH 7.4 was reached, the polypeptide formed a stable hydrogel structure after standing for five minutes. Both tilting and inverting could not alter the stable condition (Additional file 1: Fig. S9), proving that IOK polypeptide could form a stable hydrogel under neutral conditions. After drug loading, the initial state of IOK polypeptide solution appeared light grayish yellow which was relatively clear and had strong fluidity. With the increase of pH value, the polypeptide solution gradually became viscous. After the solution pH reached 7.4, the polypeptide formed a stable hydrogel structure after standing for about five minutes (Additional file 1: Fig. S10). The results showed that the drug loaded IOK peptides could also form stable hydrogels under neutral conditions (BiPM@IOK). Loading BiNS/PEI and MTX had no significant effect on the formation of pH responsive IOK peptide hydrogel. As shown in Table 1, the MTX encapsulation rate of IOK polypeptide hydrogel was about 96.91%, revealing that the designed IOK polypeptide hydrogel could enclose MTX in its dense fiber network structure which was significantly better than other drug carriers in drug encapsulation capacity.

**Table 1:** Encapsulation efficiency of drug loading IOK peptide hydrogels, n = 3

Sample	Encapsulation efficiency (%)	Mean (%)	RSD (%)
1	96.08	96.91 ± 0.84	0.87
2	96.88		
3	97.76		

**Fig. 3:**
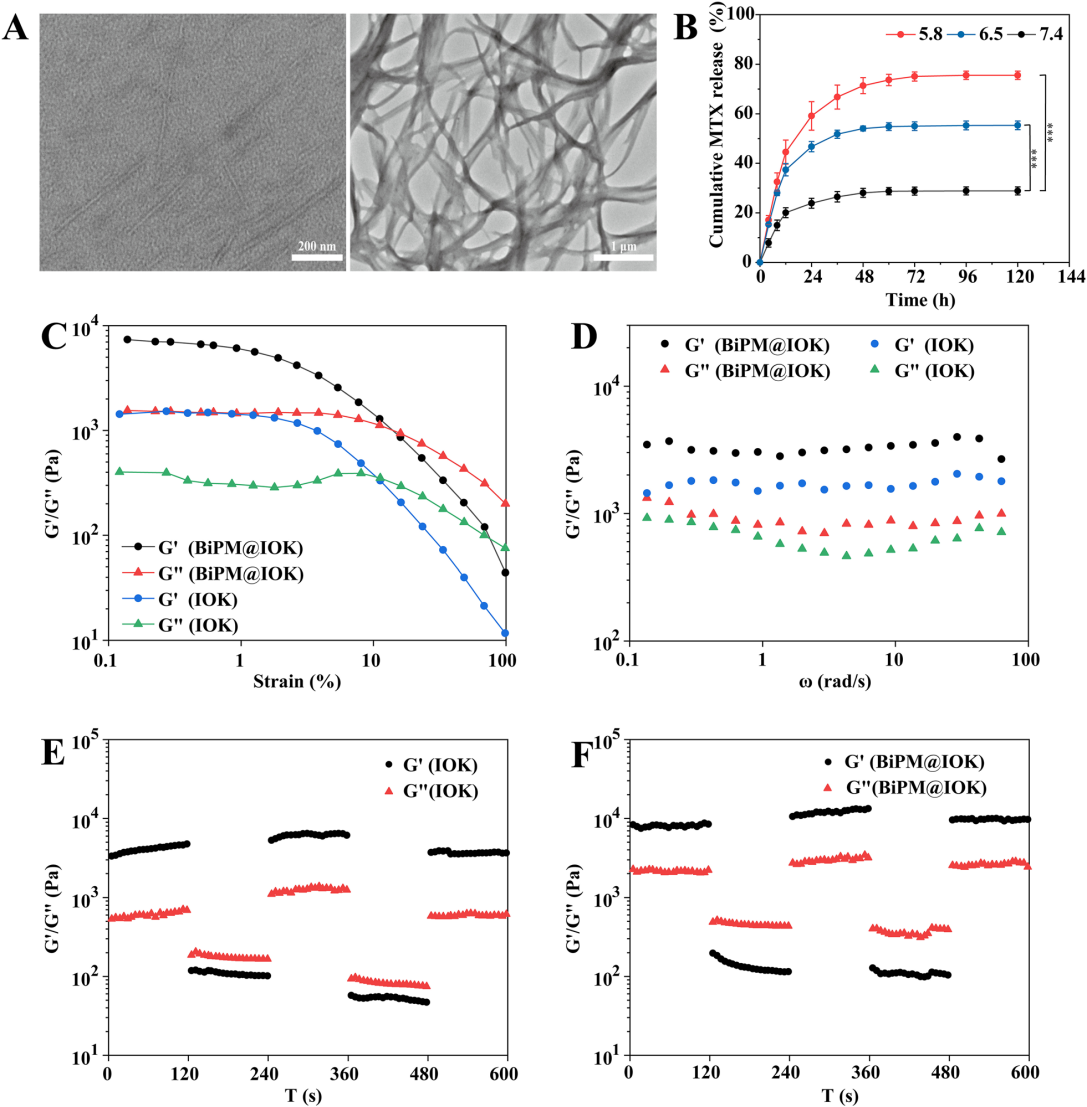
Characterization of IOK and BiPM@IOK peptide hydrogel. **A** TEM images of crushed suspension of blank IOK peptide hydrogel under pH 6.5 (Left, Scale bar: 200 nm) and pH 7.4 (Right, Scale bar: 1 μm). **B** Cumulative release rate of MTX in 120 h. The pH value of release solution was 5.8, 6.5 and 7.4, respectively, n = 3, ***P < 0.001. **C** Dynamic strain sweep of blank IOK hydrogel and BiPM@IOK hydrogel. **D** Circle sweep of blank IOK hydrogel and BiPM@IOK hydrogel. Dynamic frequency scanning of blank IOK hydrogel (**E**) and BiPM@IOK hydrogel (**F**)

The microstructure of blank IOK polypeptide hydrogels under different pH conditions was investigated by transmission electron microscope. As shown in Fig. 3A, when the ambient pH value was 7.4, IOK polypeptide fiber bundles gathered and intertwined with each other to form a dense network structure which was expedient for wrapping nanosheets and drugs. When the pH value converted to 6.5, the peptide hydrogel appeared a short fiber state of tens to hundreds of nanometers in length and dispersed in the field of vision. Under acidic conditions, the protonation of basic amino acids in IOK polypeptides carried a positive charge and the same charges repelled each other. The repulsive force between molecules was greater than the attractive force so that the polypeptide fiber was depolymerized. The change of microstructure corresponded with the transformation of polypeptide from liquid state to hydrogel state, further verifying the pH responsiveness of IOK polypeptide.

### MTX release

For examining the pH responsive release characteristics of IOK peptide hydrogel, PBS solutions with different pH values were utilized as the release solution. In RA synovium, hyperplasia of FLS and infiltration of immune cells directly lead to the acidic synovium microenvironment [49]. In our research, we set pH 7.4 to simulate the pH value of normal body fluid and pH 5.8, 6.5 as the acidic pH gradient to illustrate the drug release behavior was directly correlated with the decrease of pH in the range of 5.8–7.4.As shown in Fig. 3B, the release rate of MTX was rapid in the first 12 h and the cumulative release rate was 20.10, 37.40 and 44.55%, respectively. The release rate slowed down and eventually became flat after 72 h. Comparing the drug release curves under the three pH conditions. It can be inferred that with the decrease of pH value, the cumulative drug release rate became higher. At pH 5.8, the cumulative MTX release rate for 120 h was the highest, reaching 75.52% followed by pH 6.5 and the lowest rate was 28.86% at pH 7.4. The results showed that IOK peptide hydrogel had good pH responsiveness. Combined with TEM results, it can be deduced that with the decrease of pH value, the protonation degree of Lys and Orn in IOK polypeptide increased. The intermolecular electrostatic repulsion force would be gradually greater than the hydrophobic force and π-π accumulation force which drove the formation of hydrogel, leading to the collapse of three-dimensional network structure of IOK hydrogel and the subsequent release of MTX contained. The pH-responsive drug delivery ability of IOK polypeptide hydrogels endowed it an ideal drug delivery system at the acidic microenvironment of RA.

### Rheological properties

The rheological test evaluates the state of the sample through two parameters: the energy storage modulus and the energy dissipation modulus. The energy storage modulus G’ represents the energy stored during the elastic deformation and the energy dissipation modulus G” represents the energy lost during the viscous deformation. When G” > G’, the viscous deformation is stronger than the elastic deformation and the sample is in a fluid state. When G’ > G”, the elastic deformation is stronger than the viscous deformation and the structure of the sample has a certain rigidity and present a solid state. The dynamic strain scanning results were shown in Fig. 3C. The linear viscoelastic intervals of IOK hydrogel and BiPM@IOK hydrogel were similar, in which G’ > G” proved that the hydrogel remained solid with good mechanical strength. When the applied stress exceeded the critical value, G” > G’ proved that the hydrogel altered to a fluid state, indicating that IOK hydrogel could be flexibly altered between solid and fluid state. The critical strain value of BiPM@IOK was slightly greater than IOK, indicating that the mechanical property of the hydrogel loaded with BiNS/PEI and MTX were slightly better than that of the blank hydrogel. The possible reason was that the loading of hydrophobic drugs and nanosheets enhanced the hydrophobic force of the hydrogel, thus promoting the self-assembly of IOK peptide. The influence of frequency on the mechanical properties and stability of both hydrogels were investigated by dynamic frequency scanning. As shown in Fig. 3D, when the angular velocity ω ranged from 100 to 0.1 rad/s, the G’ of IOK hydrogels and BiPM@IOK hydrogels were consistently greater than G”, indicating that the mechanical properties of both hydrogels were not affected by frequency. At the same time, the value of G’ and G” of BiPM@IOK hydrogel were higher than that of IOK hydrogel, indicating that the rigidity of the hydrogel after drug loading was slightly stronger than blank IOK hydrogel which was consistent with the results of dynamic strain scanning. Cyclic strain time scanning experiment simulated the process of injecting the IOK hydrogel and BiPM@IOK hydrogel into the inflammation site by repeatedly applying high/low strain and investigated whether the hydrogel was stable and self-healing after being injected into synovium tissue. When a low strain of 0.1% was applied, the G’ of both samples was higher than G”, appearing solid state (Fig. 3E, F). When the stress force increased to 50%, G’ and G” both decreased significantly. At this time, G’ was less than G”, the hydrogel turned to a fluid state, that is, when a large force was applied, the hydrogel can be pushed in/out of the syringe in a fluid state. Subsequently, in another cycle, the G’ and G” of the two hydrogels were basically the same as that of the previous cycle, indicating that the peptide hydrogel could recover immediately after being exposed to greater external force. Furthermore, we recorded a video to vividly illustrate the injectable and self-healing properties. As clearly shown in Additional file 2: Video. S1, BiMP@IOK can be easily absorbed and injected by the syringe. After that, the hydrogel immediately altered from liquid to solid which was consistent with the result of rheological test. Due to its excellent self-healing properties, drug loaded IOK could be used as an ideal injectable drug delivery carrier.

### In vitro biocompatibility

**Fig. 4:**
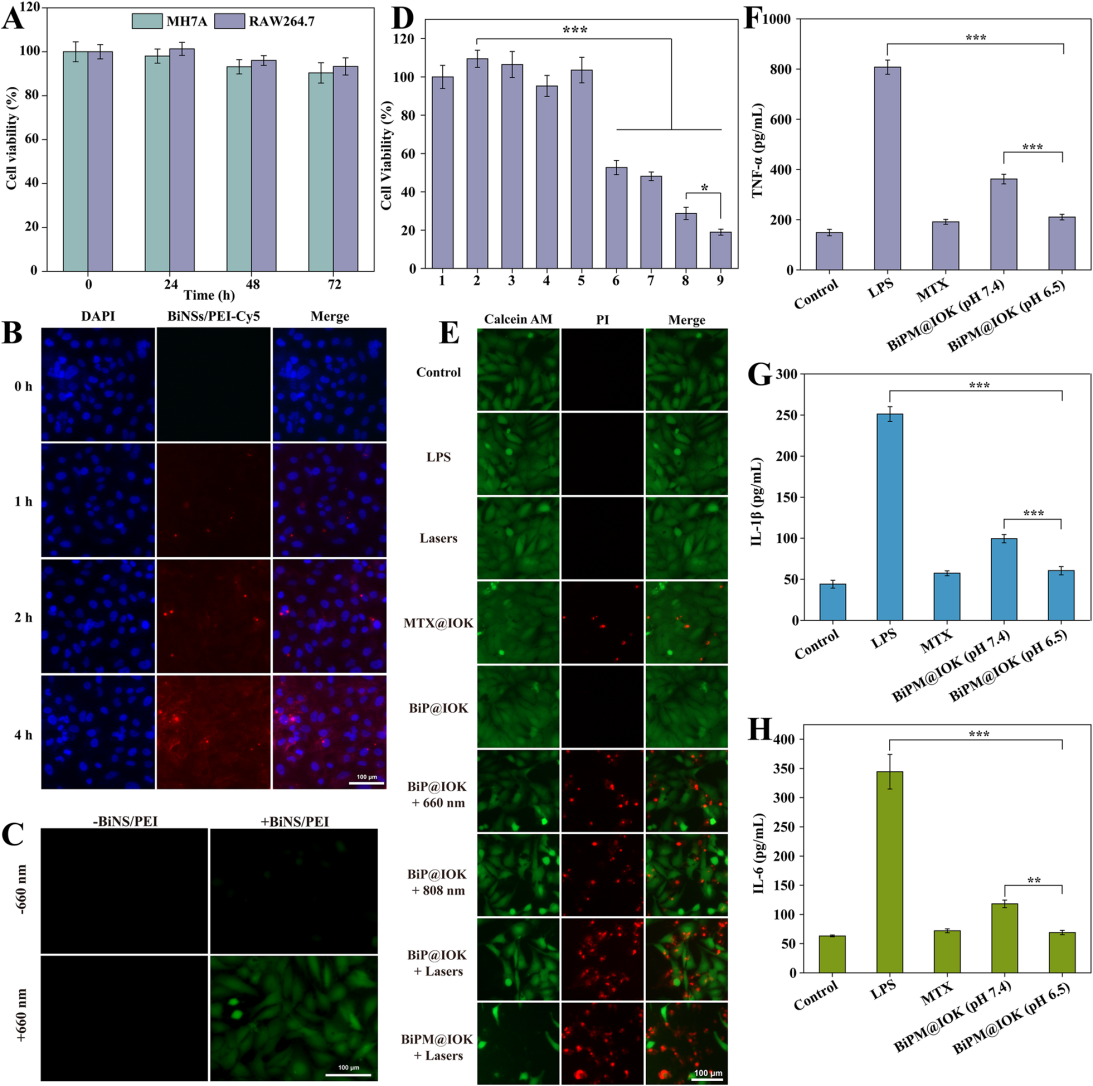
Evaluation of in vitro treatment efficacy of BiPM@IOK hydrogel. **A** Relative cell viability of MH7A and RAW264.7 incubated with BiP@IOK for 0, 24, 48 and 72 h. **B** Cellular uptake of BiNS/PEI released from BiPM@IOK labelled by Cy5-PEI. Scale bar: 100 μm. **C** Detection of intracellular ROS level stained by DCFH-DA in MH7A cells. Scale bar: 100 μm. **D** Cell viability of MH7A cells with various treatments, *, p < 0.05, ***, p < 0.001. 1: Control, 2: LPS, 3: Lasers, 4: MTX@IOK, 5: BiP@IOK, 6: BiP@IOK + 660 nm, 7: BiP@IOK + 808 nm, 6: BiP@IOK + 660 nm, 8: BiP@IOK + Lasers (660 + 808 nm), 9: BiPM@IOK + Lasers (660 + 808 nm). **E** Fluorescence images of MH7A cells co-stained by Calcein AM and PI with different treatments. Scale bar: 100 μm. Concentration of TNF-α (**F**), IL-1β (**G**) and IL-6 (**H**) secreted by RAW 264.7 cells with different treatments detected by ELISA kit, **, p < 0.01, ***, p < 0.001

Before assessing the anti-inflammatory efficacy of BiPM@IOK, ensuring the biocompatibility of the drug delivery system was essential. MH7A and RAW264.7 were incubated with BiP@IOK for 0, 24, 48 and 72 h. As shown in Fig. 4A, relative cell viabilities of two cell lines were above 90%, revealing ideal biocompatibility.

### In vitro study on clearance ability of synovial fibroblasts

The uptake of BiNS/PEI by MH7A cells was detected by Cy5 fluorescent probes. As shown in Fig. 4B, weak fluorescence signal was observed in MH7A cells after incubation with the 24 h release solution of IOK hydrogel containing BiNS/PEI-Cy5 and MTX (pH 6.5) for 1 h. The intracellular fluorescence gradually enhanced with the increase of incubation time and at the time point of 4 h, significant fluorescence appeared in the cells, indicating that BiNS/PEI-Cy5 was successfully taken up by cells. More importantly, it provided the possibility to exert PDT in MH7A cells. Then we used DCFH-DA fluorescent probe to detect ROS production to verify the photodynamic property of BiNS/PEI in cells. MH7A cells were incubated with 24 h release solution (pH 6.5) of BiPM@IOK for 4 h followed by irradiation of 0.1 W/cm^2^ 660 nm laser for 20 min. Obvious green fluorescence can be observed in the field (Fig. 4C), while neither the 660 nm laser irradiation group nor the BiNS/PEI group alone produced ROS, indicating that 660 nm laser effectively catalyzed ROS production by BiNS/PEI in MH7A cells. Subsequently, we must confirm whether the presence of MTX and PDT/PTT effect of BiNS/PEI could effectively kill over-proliferated FLS to ameliorate RA microenvironment. MH7A cells were incubated with the release solutions of different hydrogels under different light irradiation. Relative cell viability rate of each group was compared to evaluate the cell clearance ability. As shown in Fig. 4D, the inflammation model induced by LPS promoted the proliferation of MH7A cells. Free MTX could inhibit its proliferation to a certain extent, but the effect was not significant within 24 h. BiNS/PEI alone (BiP@IOK group) or 808 nm and 660 nm Lasers alone (Lasers group) had almost no cytotoxicity. By contrast, in the BiNS/PEI group irradiated with 660 nm laser or 808 nm laser (BiP@IOK + 660 nm or 808 nm group), significant cell death was observed in both the photodynamic treatment group and the photothermal treatment group alone with cell survival rate of 52.64% and 48.12%, respectively. In the combination of PDT + PTT group (BiP@IOK + Lasers group), the cell clearance ability was more significant, and the cell survival rate was 28.73%. MTX combined with light therapy had the best cell clearance efficacy (BiPM@IOK + Lasers) with a cell survival rate of only 18.90%. In general, BiNS/PEI has excellent intracellular photothermal and photodynamic property. When combined with MTX, a drug commonly used in the clinical treatment of RA, BiNS /PEI can remarkably remove the excessive proliferation of FLS in the pathological microenvironment. To further verify the cell clearance efficacy of BiPM@IOK on MH7A cells in vitro, we conducted Calcein-AM/PI live and dead cell double staining experiment. As shown in Fig. 4E, a small amount of red fluorescence was observed in the MTX@IOK group, indicating that MTX had a weak scavenging ability on MH7A cells within 24 h. Strong green fluorescence signal was observed in the BiP@IOK and bare Lasers groups as BiNS/PEI alone or lasers alone had no killing effect on cells. In the BiP@IOK + 660 nm laser and BiP@IOK + 808 nm laser irradiated groups, obvious red fluorescence was observed. The red fluorescence of BiP@IOK + Lasers was much stronger than that of the above two groups. The combination of PDT and PTT significantly enhanced the cell clearance effect. The red fluorescence of BiPM@IOK + Lasers was strongest so that it had the best cell clearance effect under laser irradiation. The conclusions were consistent with the results of cytotoxicity experiments.

### In vitro anti-inflammatory efficacy

Macrophage played the pivotal role in secreting pro-inflammatory cytokines such as TNF-α, IL-1β and IL-6 in RA so we quantitatively measured the levels of cytokines secreted by RAW 264.7 cells to evaluate the alleviating effect of MTX on inflammation. As shown in Fig. 4F–H, the concentration of three pro-inflammatory cytokines in the LPS-induced inflammation model group remarkably increased to almost 5 times of the control group. Free MTX (500 ng/mL) had a superb anti-inflammatory effect on RAW 264.7 cells and three cytokines downregulated to the level of control group. After being incubated with the release solution of BiPM@IOK with the pH value of 7.4 and 6.5, cytokine levels in both groups significantly decreased. Predictably, the level of cytokines in the pH 7.4 group after 24 h incubation was higher than that of the pH 6.5 group as the collapse of internal network of IOK hydrogel was more complete under inflammatory acidic conditions and more MTX was released. After detection, MTX in the release solution of pH 7.4 and pH 6.5 was 231.26 ng/mL and 486.51 ng/mL.

### In vivo distribution

**Fig. 5:**
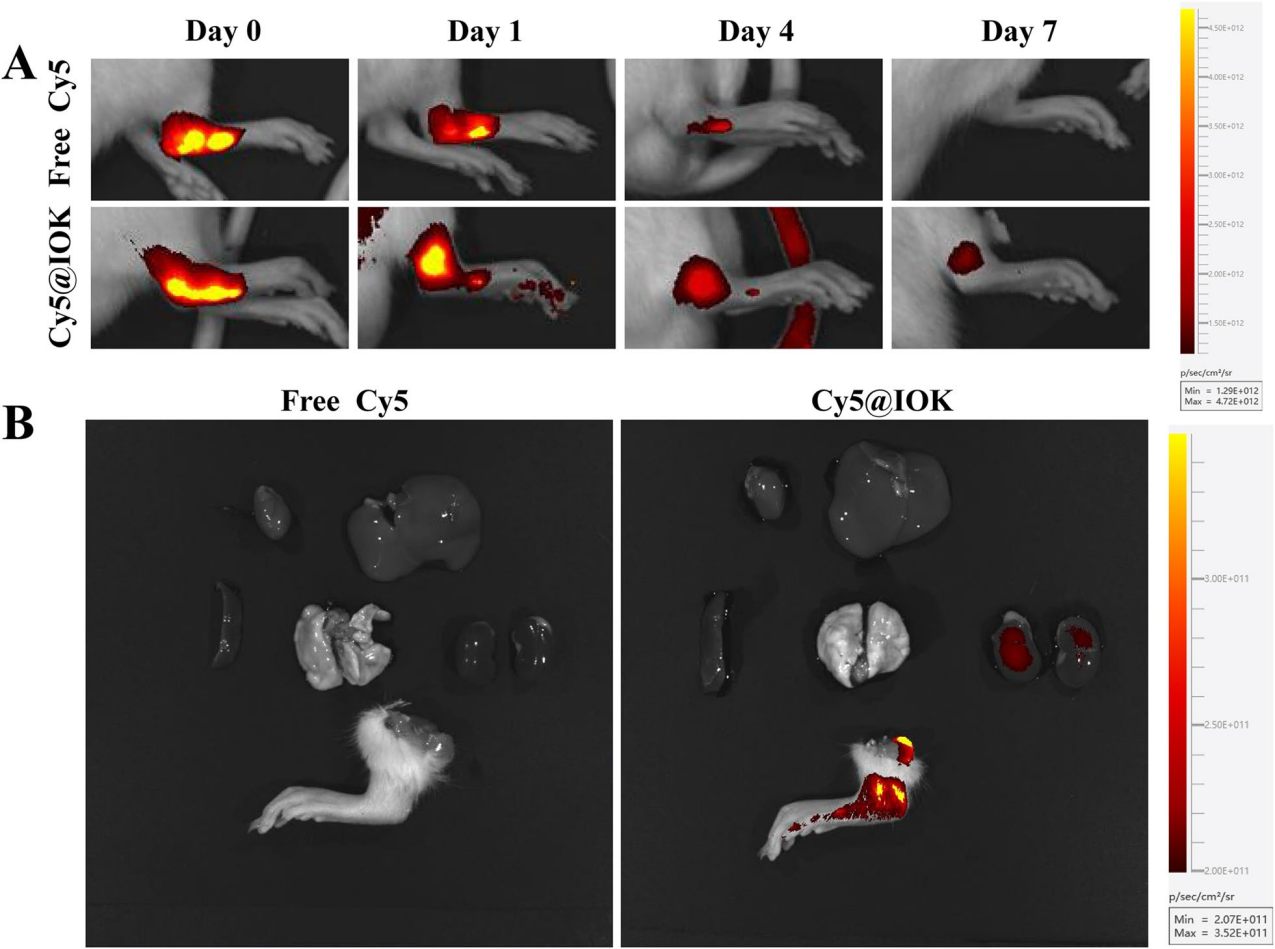
Biodistribution of Free Cy5 and Cy5@IOK in vivo. **A** In vivo fluorescence images of AIA rats. The images were recorded at Day 0, 1, 4, 7. **B** Ex vivo fluorescence images of right hind paws and major organs dissected from AIA rats

For investigating the biodistribution of BiPM@IOK hydrogel after local injection in synovium tissues, fluorescence imaging analysis was performed on rats injected with Cy5 fluorescent probe solution and Cy5@IOK hydrogel within 7 days. As shown in Fig. 5A, fluorescence intensity in the synovium of Free-Cy5 group rapidly weakened within 4 days and the fluorescence signal cannot be observed on Day7, illustrating that Cy5 was metabolized in a short time. The decrease rate of fluorescence intensity in Cy5@IOK group was much slower than Free Cy5 group, indicating that the ability of hydrogel to retain drugs was much stronger than free drugs. 7 days after administration, the main organs (heart, liver, spleen, lung, kidney) and right posterior feet were dissected. Fluorescence signals of the Free-Cy5 group completely disappeared (Fig. 5B). Fluorescence intensity of Cy5@IOK group concentrated in the synovium, indicating that IOK peptide hydrogel, as a local drug reservoir, could accumulate drugs at the administration site and play the role in controlled and sustained drug release. Meanwhile, the fluorescence signal can also be observed in kidneys due to rapid renal metabolism of Cy5.

### In vivo anti-rheumatoid arthritis study

**Fig. 6:**
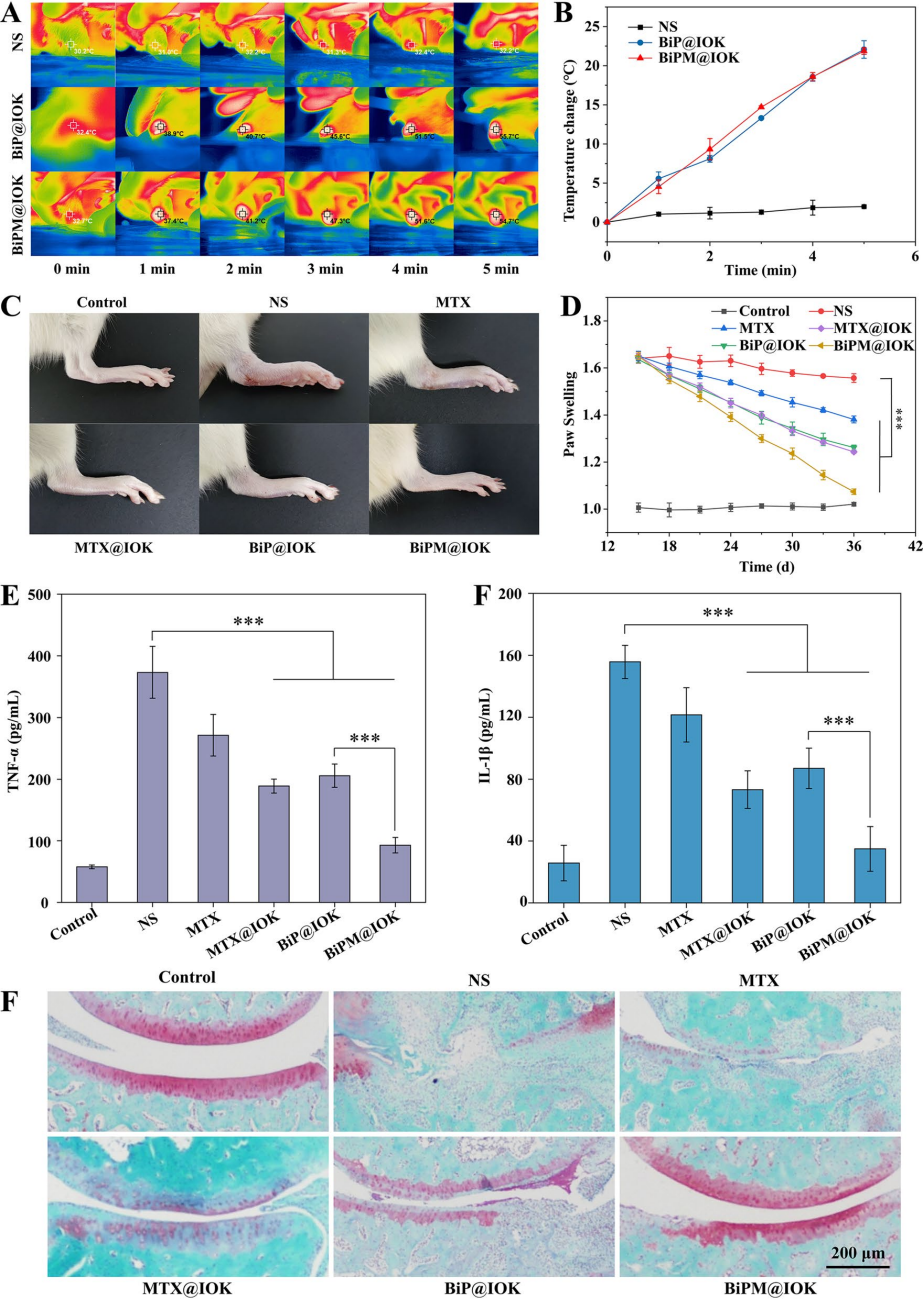
In vivo treatment efficacy of BiPM@IOK hydrogel. **A** Representative infrared thermal images of right hind ankle joints of AIA rats with different treatments under NIR (808 nm, 1 W/cm^2^) irradiation for 5 min. **B** Temperature increase curves according to (**A**). Representative images of right hind paws (**C**) and paws swelling degree (**D**) of normal and AIA rats with different treatments, ***, p < 0.001. Concentration of TNF-α (**E**) and IL-1β (**F**) of synovium tissue samples detected by ELISA kit, ***, p < 0.001. **G** Safranin O staining of joints after various administrations

The photothermal properties of BiNS/PEI in vitro have been investigated previously. In this part, we firstly investigated the photothermal properties of BiNS/PEI in vivo by observing the temperature changes in synovium. As shown in the typical photothermal images in Fig. 6A, the synovium temperature of AIA rats injected with normal saline showed no significant change within 5 min irradiated by 1 W/cm^2^ 808 nm near infrared light. Temperature of the synovium injected with BiP@IOK and BiPM@IOK hydrogel rapidly increased and reached 55.7 and 54.7 ℃ after 5 min, respectively. According to the curve of synovium temperature (Fig. 6B), the temperature change of BiP@IOK and BiPM@IOK group was 22.07 and 21.87 ℃ within 5 min, respectively. The results showed that BiNS/PEI had good photothermal conversion effect in vivo which would ablate FLS to treat RA.

Then we investigated the anti-inflammatory efficacy of BiPM@IOK and AIA model was constructed for following research. Healthy rats were taken as negative control group and AIA rats with NS injected locally into the synovium were taken as positive control group. Changes in the degree of synovium swelling in different treatment groups were observed. IOK hydrogel alone was not discussed as IOK peptide could not reverse the inflammation induced by LPS (Additional file 1: Fig. S13). Meanwhile, IOK hydrogel system cannot lead to obvious cell death (Fig. 4A) like PDT and PTT therapy of BiNS/PEI. We believed that IOK hydrogel alone would have no influence on the treatment efficiency of RA. As shown in Fig. 6C, the joint synovium swelling of NS group was the most serious, showing a state of swelling or even fester at the inflammatory site. The paw swelling of AIA rats in Free MTX group was slightly relieved, but the overall efficacy was poor as free MTX was hard to retain in synovium according to the in vivo imaging experiment. Compared with the two groups, MTX@IOK and BiP@IOK had nearly the same therapeutic efficacy and the paw swelling was significantly reduced in both groups. MTX@IOK directly inhibited activity of various immune cells which secrete pro-inflammatory cytokines and chemokines. BiP@IOK reversed hyperplasia of FLS via PDT and PTT routes as FLS played the central role in synovium inflammation through secreting cytokines/chemokines, inducing cartilage/bone damage and creating hypoxia synovium microenvironment. The elimination of FLS profoundly ameliorate synovium microenvironment. The ankle joint of BiPM@IOK group had almost no symptoms of redness and swelling. The degree of swelling was the mildest as well. We used the ratio of the thickness of the right and left hind paws to indicate the degree of synovium swelling in each group (Fig. 6D). The analysis results were consistent with the image of paws showed in Fig. 6C. The BiPM@IOK hydrogel combining PDT, PTT and drug therapy had the best curative effect in reducing synovium swelling. Finally, we analyzed the levels of TNF-α and IL-1β in synovium tissue by ELISA assay. As shown in Fig. 6E, F, the anti-inflammatory efficacy of free MTX group was significantly worse than that of MTX@IOK group, indicating that IOK polypeptide hydrogel served as a delivery carrier and drug repository to achieve controlled release at the inflammatory site and achieve better drug therapeutic effect. Compared with NS group, MTX@IOK group, BiP@IOK group and BiPM@IOK group significantly reduced the levels of TNF-α and IL-1β in synovium tissue of rats. The BiPM@IOK group had the best anti-inflammatory efficacy and the levels of TNF-α and IL-1β in the synovium were close to those in Control group, indicating that the combination of drug therapy and phototherapy could act on FLS and macrophages in a two-prong manner to inhibit synovium inflammation. Finally, Safranin O staining was performed to evaluate whether BiPM@IOK could remedy cartilage damage induced by RA. Cartilage in the control group remained smooth and intact (Fig. 6G). After AIA administration, the cartilage tissue almost disappeared due to severe inflammatory phenotype; BiP@IOK and MTX@IOK obviously rescued this phenomenon. With the irradiation of 660 and 808 nm lasers, BiP@IOK eliminated FLS to reduce the release of MMPs which was responsible for cartilage erosion while MTX@IOK reduced activity of various cells in synovium, delaying the process of RA and corresponding cartilage erosion. Not surprisingly, BiPM@IOK achieved the best cartilage protection efficacy which was consistent with previous results.

Pathological studies of major organs were conducted to investigate whether in situ injection BiPM@IOK for 3 weeks had any damage to the organs. H&E staining results of various organ sections were shown in Additional file 1: Fig. S14. No obvious inflammation or lesions were found in the heart, liver, spleen, lung and kidney of rats in BiPM@IOK group compared with those in NS group. Besides, the levels of RBC, WBC, ALT, AST, CRE and BUN had no significance between NS group and BiPM@IOK group (Additional file 1: Fig. S15), noting that BiPM@IOK had good biocompatibility in vivo.

## Conclusions

In this research, we successfully developed a pH sensitive and injectable peptide hydrogel loading BiNS/PEI and antirheumatic drug MTX for the effective treatment of RA. The BiPM@IOK peptide was injected into inflammatory synovium to enhance targeting ability and sustained drug release. Acidic RA microenvironment promoted protonation of IOK peptide due to the presence of alkaline amino acid Orn/Lys and PEI so that the electrostatic repulsion increased rapidly, leading to the collapse of peptide hydrogel and the accelerated drug release. The released BiNS/PEI and MTX were directly absorbed by various cells in the synovium. MTX significantly reduced TNF-α, IL-1β and IL-6 secreted by macrophages which play the pivotal role in releasing pro-inflammatory cytokines, attenuating the symptoms of RA instantly. The apply of BiNS/PEI precisely overcame the obstacles of FLS clearance, 808 nm and 660 nm laser induced PTT and PDT combined MTX effectively eliminated activated FLS which serve as the central role in RA through secreting pro-inflammatory cytokines, chemokines, MMPs and creating hypoxia microenvironment. We believed that our BiPM@IOK drug delivery system was designed for severe RA accompanied by hyperplasia of FLS, and ideal treatment efficacy has been achieved both in *vitro and *in vivo.

### Supplementary Information

**Additional file 1: Fig S1.** XRD patterns of Bi powder and BiNS and the corresponding standard patterns of Bi (PDF#85-2329) and Bi_2_O_3_ (PDF#71-2274). **Fig S2.** TEM and EDS mapping images of BiNS. Scale bar: 25 nm. **Fig S3.** Particle size of BiNS/PEI within 7 days at 4, 25 and 37℃. **Fig S4.** Images of BiNS suspended in ethanol(A), lyophilized BiNS powder re-suspended in water without (B) or with (C) ultrasonication, BiNS/PEI in water (D), BiNS/PEI stored in water for 3 months (E). **Fig S5.** Synthesis route of designed peptide on Wang resin. **Fig S6.** Morphology of IOK peptide. **Fig S7.** High performance liquid chromatography of IOK peptide. **Fig S8.** Mass spectrum of IOK peptide. **Fig S9.** Morphology of blank IOK hydrogel before (A) and after (B) pH adjusting. **Fig S10.** Morphology of BiPM@IOK hydrogel before (A) and after (B) pH adjusting. **Fig S11.** UV-vis-NIR absorption spectra of IOK peptide and MTX. **Fig S12.** Standard curve of MTX in PBS solution. A = 0.0469x + 0.0081，R^2^ = 0.9991. **Fig S13.** Concentration of TNF-α (A), IL-1β (B) and IL-6 (C) secreted by RAW 264.7 cells treated with LPS and LPS+IOK. n.s.: no significance. **Fig S14.** H&E-staining images of main organs dissected from rats with local synovium injection of NS or BiPM@IOK. Scale bar: 50 μm. **Fig S15.** Hematological parameters (A) RBC, (B)WBC and serum levels of (C) ALT, (D) AST, (E) BUN, (F) CR rats collected from treated with Normal Saline or BiPM@IOK. n=5, n.s: no significance. **Table S1.** Chromatography condition of HPLC

**Additional file 2: Video S1.** Absorbing, injecting and recovering process of BiP@IOK

## Data Availability

Data will be available on request.
