# Supplementary material for: Synovium microenvironment-responsive injectable hydrogel inducing modulation of macrophages and elimination of synovial fibroblasts for enhanced treatment of rheumatoid arthritis
Source: J Nanobiotechnology. 2024 Apr 17;22:188. doi: 10.1186/s12951-024-02465-w (PMC11025172; doi:10.1186/s12951-024-02465-w)
Supplement: Supplementary file 1 — Additional file 1: Fig S1. XRD patterns of Bi powder and BiNS and the corresponding standard patterns of Bi (PDF#85-2329) and Bi2O3 (PDF#71-2274). Fig S2. TEM and EDS mapping images of BiNS. Scale bar: 25 nm. Fig S3. Particle size of BiNS/PEI within 7 days at 4, 25 and 37℃. Fig S4. Images of BiNS suspended in ethanol(A), lyophilized BiNS powder re-suspended in water without (B) or with (C) ultrasonication, BiNS/PEI in water (D), BiNS/PEI stored in water for 3 months (E). Fig S5. Synthesis route of designed peptide on Wang resin. Fig S6. Morphology of IOK peptide. Fig S7. High performance liquid chromatography of IOK peptide. Fig S8. Mass spectrum of IOK peptide. Fig S9. Morphology of blank IOK hydrogel before (A) and after (B) pH adjusting. Fig S10. Morphology of BiPM@IOK hydrogel before (A) and after (B) pH adjusting. Fig S11. UV-vis-NIR absorption spectra of IOK peptide and MTX. Fig S12. Standard curve of MTX in PBS solution. A = 0.0469x + 0.0081，R2 = 0.9991. Fig S13. Concentration of TNF-α (A), IL-1β (B) and IL-6 (C) secreted by RAW 264.7 cells treated with LPS and LPS+IOK. n.s.: no significance. Fig S14. H&E-staining images of main organs dissected from rats with local synovium injection of NS or BiPM@IOK. Scale bar: 50 μm. Fig S15. Hematological parameters (A) RBC, (B)WBC and serum levels of (C) ALT, (D) AST, (E) BUN, (F) CR rats collected from treated with Normal Saline or BiPM@IOK. n=5, n.s: no significance. Table S1. Chromatography condition of HPLC [file 12951_2024_2465_MOESM1_ESM.docx]

**Synovium Microenvironment-responsive Injectable Hydrogel Inducing Modulation of Macrophages and Elimination of Synovial Fibroblasts for Enhanced Treatment of Rheumatoid Arthritis**

Yiqun Wu^1, ‡^, Yu Ge^1, ‡^, Zhongshi Wang^1,5 ‡^, Ying Zhu^6^, Tianli Tian^1^, Jun Wei^1^, Yu Jin^1^, Yi Zhao^6^, Qiang jia^7^, Jun Wu^2,3,4,^ *, Liang Ge^1,^ *

*^‡^* These authors contributed equally to the work

* Co-corresponding authors

^1^State Key Laboratory of Natural Medicines, School of Pharmacy, China Pharmaceutical University, Nanjing, Jiangsu, 210009, China.

^2^Guangdong Provincial Key Laboratory of Malignant Tumor Epigenetics and Gene Regulation, Sun Yat-sen Memorial Hospital, State Key Laboratory of Oncology in South China, Guangzhou, 510120, China.

^3^Bioscience and Biomedical Engineering Thrust, The Hong Kong University of Science and Technology (Guangzhou), Nansha, Guangzhou, 511458, China.

^4^Division of Life Science, Hong Kong University of Science and Technology, Hong Kong SAR, 999077, China.

^5^Department of Pharmacy, The Affiliated Hospital of Nantong University, Jiangsu, 226006, China.

^6^Department of Pharmacy, The First Affiliated Hospital of Soochow University, Suzhou, Jiangsu, 215026, China.

^7^School of Biomedical Engineering, Shenzhen Campus of Sun Yat-sen University, Shenzhen 518107, China.

^8^Guangzhou City Polytechnic, Guangzhou, Guangdong, 510520, China.

Corresponding author:

Liang Ge, E-mail address: Geliang1981@hotmail.com.

Jun Wu, E-mail address: junwuhkust@ust.hk.

**Experimental sections**

**Materials**

Bi powder was purchased from Aladdin; MTX and Cy5 were purchased from Shanghai Yuanye Bio-Technology Co., Ltd; PEI 10 k, TEMP, DMPO and DPBF were purchased from Sigma Alrich; RAW 264.7 cell was purchased from ATCC; MH7A cell was purchased from Mingzhou Bio; DCFH-DA probe was purchased from Solarbio; CCK8 kit and Calcein-AM/PI reagent were purchased from Shanghai Yeasen; SD rats were purchased from Nanjing Qinglongshan Animal Centre; Complete Freund’s Adjuvant (1 mg/mL) was purchased from Chondrex; ELISA kits for murine were purchased from Abclonal; ELISA kits for rat were purchased from Abcam.

**Preparation of BiNS**

300 mg Bi powder was placed in 60 mL anhydrous ethanol and sonicated by ultrasonic probe in ice bath for 12 h (Parameters: power 500 W, ultrasonic time 2 s, interval 1 s). Subsequently, the suspension was centrifuged at 12000 rpm for 30 min and the supernatant was removed. 15 mL deionized water was added to suspend Bi powder and the suspension was frozen at -80℃ overnight. After thawing on the next day, the suspension was centrifuged at 12000 rpm for 30 min at 4℃ and the supernatant was removed. The collected precipitation was placed in 60 mL ethanol for another sonication with the same parameters mentioned above. After ultrasonication, the suspension was centrifuged at 5000 rpm for 10 min to remove large pieces of Bi powder. The collected supernatant was centrifuged at 12000 rpm for 40 min. The precipitation was collected and quickly suspended with 5 mL deionized water (pre-blown nitrogen to remove oxygen from the water). After freeze-drying, the product BiNS was obtained and stored in a vacuum dryer.

**Preparation of BiNS/PEI**

2 mg 10 kDa PEI was dissolved in 10 mL PBS (pH 7.2-7.4). Subsequently, 5 mg BiNS was dispersed in PEI solution and sonicated by the probe with power of 200 W for 30 min under the condition of dark and ice bath. Subsequently, the suspension was stirred for 8 h and centrifuged at 12000 rpm at 4℃ for 40 min to remove excess PEI. The collected BiNS/PEI was re-suspended with deionized water and stored at 4℃.

**TEM of BiNS and BiNS/PEI**

BiNS or BiNS/PEI was dropped on the surface of the copper mesh and dried by infrared lamp. After that, the samples were placed into the transmission electron microscope and the morphology of BiNS or BiNS/PEI was observed and recorded.

**Particle size and Zeta potential of BiNS and BiNS/PEI**

BiNS was re-suspended in ethanol and BiNS/PEI was suspended with deionized water. Both samples were dispersed uniformly by probe ultrasonication. The particle size distribution and Zeta potential was tested.

**Study on the photothermal properties of BiNS/PEI**

In the concentration gradient experiment, BiNS/PEI with concentrations of 0, 25, 50, 100 and 200 μg/mL were prepared. The suspension was irradiated by 808 nm near-infrared light with the power of 1.0 W/cm^2^ for 10 min.

In the power gradient experiment, 100 μg/mL BiNS/PEI was irradiated by 0.5, 1.0 and 1.5 W/cm^2^ 808 nm laser for 10 min, respectively. The temperature change of the suspension was recorded by an infrared thermal imager every 0.5 min.

In order to verify the photothermal stability of BiNS/PEI, "on/off cycle" experiment was carried out. 1 mL 100 μg/mL BiNS/PEI was irradiated with 808 nm near-infrared light with the power of 1.0 W/cm^2^ at room temperature for 5 min. Then the sample was cooled to room temperature naturally. 3 cycles were performed totally.

**DPBF probe**

3 mL 100 μg/mL BiNS/PEI was placed in 5 mL brown cillin bottle after being fully dispersed by ultrasonication. 2.7 mg DPBF probe was dissolved in 2 mL DMSO. 120 μL of the DPBF solution was dissolved in the BiNS/PEI suspension and stirred for 15 min. The sample was irradiated by 660 nm laser with the power of 0.1 W/cm^2^ for 30 min in dark. The absorption spectrum of the sample at different time points (0, 5, 10, 15, 20, 25, 30 min) were recorded by ultraviolet/visible spectrophotometer.

**Electron spin resonance**

Superoxide anion (O_2_^·-^) was detected in methanol system using 5, 5-dimethyl-1-pyrrolin-n-oxide (DMPO) as the spin catcher and singlet oxygen (^1^O_2_) was detected using 4-amino-2, 2, 6, 6-tetramethylpiperidine (TEMP) as the spin catcher. BiNS/PEI suspension and TEMP/DMPO were mixed in a sealed container, pumped with air and irradiated with 0.1 W/cm^2^ 660 nm laser for 20 min and ESR signals were recorded.

**Synthesis and characterization of IOK polypeptides**

Fmoc/tBu orthogonal protection method was adopted and the synthesis were shown in Fig. S5. Using Wang resin as solid phase carrier. The C end of the polypeptide chain was fixed on the resin and the amino acids were dehydrated and condensed to form peptide bonds in sequence from C end to N by using condensation mixture (DIC and HOBt) until the complete peptide chain was synthesized. Finally, the lysate (TFA: TIS: EDT: H_2_O = 95:2:2:1) was added to cut the polypeptide chain and IOK peptide was prepared. The purity of IOK polypeptide was analyzed by high performance liquid chromatography and the structure was confirmed by mass spectrometry. The HPLC conditions were shown in Tab. S1.

**Preparation of blank IOK and drug loaded BiPM@IOK hydrogel**

2.0 mg IOK polypeptide was placed in 100 μL 150 mM NaCl and 20 mg/mL peptide solution was obtained. The pH of the solution was adjusted to 7.4 with NaOH and HCl. The blank IOK hydrogel was obtained within 5 min.

2.0 mg IOK polypeptide was placed in 100 μL 0.5 mg/mL MTX and 2 mg/mL BiNS/PEI suspension which was diluted by 150 mM NaCl. The drug loaded BiPM@IOK hydrogel was obtained with the same method.

**MTX standard curve**

2.5 mg MTX was diluted by PBS with the pH 7.2-7.4 in a 50 mL brown volumetric flask and 5 mg/mL MTX liquor was achieved. The MTX liquor was diluted by PBS to obtain MTX solution with the concentration of 1, 2, 5, 10, 12, 15 and 20 μg/mL. The absorbance of the above MTX solutions were measured by ultraviolet/visible spectrophotometer at 304 nm wavelength (Fig. 10). The standard curve (Fig. S12) was drawn with the concentration of MTX as the horizontal coordinate and the absorbance as the vertical coordinate.

**Study on the encapsulation rate and in vitro release of MTX**

200 μL BiPM@IOK hydrogel (MTX: 0.5 mg/mL, BiNS/PEI: 2 mg/mL, IOK peptide: 20 mg/mL) were prepared in parallel. The surface hydrogel was gently rinsed with 2 mL PBS (pH 7.4) buffer and the encapsulation rate of the MTX was calculated.

To further verify the pH-responsive drug release properties of IOK polypeptide hydrogel, MTX release was studied *in vitro*. 200 μL BiPM@IOK hydrogel were prepared in parallel. Surface of the hydrogel was gently rinsed with 2 mL pH 7.4 PBS buffer. After that, 2 mL pH 5.8, pH 6.5 and pH 7.4 PBS buffer were added to the gel surface of each group respectively as the release solution. The EP tube was placed in a constant temperature shaking table (120 rpm, 37℃). At specific time points (4, 8, 12, 24, 36, 48, 60, 72, 96, 120 h), the release solution was collected, and the same volume of PBS was added. The release solution was centrifuged at 12000 rpm for 40 min to remove BiNS/PEI then filtered by 0.22 μm microporous filter membrane. The absorbance of each release solution was measured and the release amount of MTX was calculated. The release curve of MTX released by hydrogel under different pH conditions was drawn.

Encapsulation rate (%) = (MTX _input_ - MTX _content in recovery liquid_)/MTX _input_ ×100%

**Hydrogel rheological detection**

To evaluate the mechanical properties of IOK hydrogel, rheological tests were performed with the HAAKE rotary rheometer. Blank IOK and drug loaded BiPM@IOK hydrogel were prepared. The test temperature was set at 37℃.

Dynamic strain scanning: scanning frequency was 1 Hz (angular velocity 6.283 rad/s); strain range was 0.1%-100%. The strain scanning was performed to evaluate the linear viscoelastic region and stability of the sample.

Dynamic frequency scanning: scanning strain value was 0.1%; frequency range was 100 rad/s-1 rad/s for frequency scanning.

Cyclic strain scanning: scanning frequency is 1 Hz (angular speed 6.283 rad/s) and the period of one cycle was set to 120 s. The strain of the first cycle was 0.1% and then increased to 50% in the second cycle. Five cycles were performed totally.

**Biocompatibility**

The biocompatibility was evaluated by Cell Counting Kit-8 reagents. MH7A cells and RAW 264.7 cells were seeded in the 96-well plate with the density of 5×10^3^ cells/well. The cells were cultured in a 37℃, 5% CO_2_ incubator for 24 h. Release medium of BiP@IOK for 0, 24, 48 and 72 h were incubated with cells for 0, 24, 48 and 72 h, respectively. After incubation, 10 μL CCK-8 reagent was added and incubated for another 2 h. OD value was measured at wavelength 450 nm. Relative cell viabilities were calculated according to the following formula:

$$\text{Cell survival rate =}\frac{\text{OD}_{\mathrm{target}}\text{-}\text{OD}_{\mathrm{blank}}}{\text{OD}_{\mathrm{control}}\text{-}\text{OD}_{\mathrm{blank}}}\text{×100\%}$$

**Cell uptake**

BiNS/PEI-Cy5 and corresponding BiP-Cy5M@IOK hydrogel were prepared. BiP-Cy5M@IOK hydrogel were cultured with pH 6.5 DMEM as the release solution. The solution was collected after 24 h. MH7A cells were seeded into 24-well plate and incubated for 24 h. Then the old medium was discarded and 500 μL 24 h release solution (pH 6.5) was added to each well and incubated for 0, 1, 2 and 4 h, respectively. Each well was cleaned with PBS for 3 times. The cells were fixed at room temperature for 40 min with 4% paraformaldehyde. The nuclei were stained with DAPI (5 μg/mL) for 5 min. MH7A cell uptake of BiNS/PEI nanoparticles was observed and recorded by inverted fluorescence microscope.

**Intracellular ROS detection.**

ROS production of BiNS/PEI under 660 nm laser irradiation was investigated by ROS probe (DCFH-DA). Experimental groups were set as: (1) Control group; (2) BiNS/PEI group; (3) 660 nm laser group; (4) BiNS/PEI + 660 nm laser group. MH7A cells were seeded into 24-well plates, (1) and (3) were added with 500 μL blank DMEM, (2) and (4) were added with 500 μL BiPM@IOK 24 h release solution. After incubating for 4 h, the medium was discarded. Each well was cleaned with PBS for 3 times. DCFH-DA probe (10 μM) was added and continued to incubate in the dark for 20 min. (3) and (4) were irradiated with red light with 0.1 W/cm^2^ 660 nm laser for 20 min. After PBS cleaning for 3 times, fresh medium was added. Fluorescence images of each group were observed by inverted fluorescence microscope.

**Cytotoxicity of BiPM@IOK**

MTX@IOK hydrogel (MTX: 10 μg/mL), BiP@IOK hydrogel (BiNS/PEI: 2 mg/mL), BiPM@IOK hydrogel (BiNS/PEI: 2 mg/mL, MTX: 10 μg/mL) were prepared. The pH of DMEM medium was adjusted to 6.5 and used as the release solution. After 24 h of release, the release solution was collected for use.

MH7A cells were seeded into 96-well plate and 9 groups were set up. 100 μL DMEM medium was added to each hole. Lipopolysaccharides (LPS) (1 μg/mL) was added to each group except Control group to induce MH7A cells to activate NF-κB and other inflammatory signaling pathways. The experimental groups were as followed: (1) Control group: blank DMEM; (2) LPS group: blank DMEM + 1 μg/mL LPS; (3) Lasers group: blank DMEM (1 μg/mL LPS) and 808 nm laser for 5 min and 660 nm laser for 20 min; (4) MTX@IOK group: 24 h release solution of MTX@IOK; (5) BiP@IOK group: 24 h release solution of BiP@IOK; (6) BiP@IOK + 660 nm group: 24 h release solution of BiP@IOK and 660 nm laser for 20 min; (7) BiP@IOK + 808 nm group: 24 h release solution (pH 6.5) of BiP@IOK and 808 nm laser for 5 min; (8) BiP@IOK + Lasers group: 24 h release solution of BiP@IOK, 808 nm laser for 5 min and 660 nm laser for 20 min. (9) BiPM@IOK + Lasers group: 24 h release solution of BiPM@IOK, 808 nm laser for 5 min and 660 nm laser for 20 min. With the same volume of DMEM as the blank control, the survival rate of each group was measured by CCK-8 method after 24 h.

The cells in each group were stained with Calcein-AM/PI. Calcein-AM was used to label living cells with green fluorescence and PI was used to label dead cells with red fluorescence. After the cells in each group were treated for 24 h, each well was incubated with Calcein-AM/PI reagent (Calcein-AM: 2 μM, PI: 4.5 μM) for 15 min. The fluorescence images were observed by inverted fluorescence microscope.

**BiPM@IOK *in vitro* anti-inflammatory effect**

RAW 264.7 cells were seeded into 24-well plate. The experimental group was set as (1) Control group: blank DMEM; (2) LPS group: DMEM + 1 μg/mL LPS; (3) MTX group: DMEM + free MTX (0.5 μg/mL); (4) BiPM@IOK (pH 7.4) group: 24 h release solution (pH 7.4) of BiPM@IOK; (5) BiPM@IOK (pH 6.5) group: 24 h release solution (pH 6.5) of BiPM@IOK. Except group (1), LPS (1 μg/mL) were added to the medium of each group during the whole experiment. After 24 h, the concentrations of TNF-α, IL-6 and IL-1β were detected by ELISA.

**Construction of AIA rat model of rheumatoid arthritis**

Adjuvant-induced arthritis (AIA) model was used in this research. After feeding (180 ± 20) g male SD rats for one week, 100 μL Freund's complete adjuvant (FCA) (1 mg/mL) was injected into the foot pad of the rat's right hind paw to induce rheumatoid arthritis. Then, the foot thickness was observed and measured every 3 days. The degree of joint synovium swelling was assessed. On the 8th day, FCA was injected with half dose to strengthen the immunization for another week.

***In vivo* imaging experiment**

The distribution of BiNS/PEI and MTX contained in BiPM@IOK hydrogel in AIA rats was studied by animal vivo imager. Cy5 fluorescent probe was implanted into IOK hydrogel in place of BiNS/PEI and MTX. AIA model rats were randomly divided into free-Cy5 group and Cy5-loaded hydrogel (Cy5@IOK) group. In the two groups, 100 μL Cy5 solution and 100 μL Cy5@IOK hydrogel (Cy5: 1 mg/kg) were injected into the synovium of the right posterior feet joint of AIA rats, respectively. Rats were anesthetized by intraperitoneal injection of 20% uratane solution (1 g/kg) 0, 1, 3, 5 and 7 days after administration. The rats were placed in the *in vivo* imager for imaging analysis. The main organs (heart, liver, spleen, lung, kidney) and right feet of rats were collected to analyze the distribution of Cy5 probes.

**Photothermal study *in vivo***

To investigate the photothermal properties of BiNS/PEI in AIA model rats, 14 days after the initial injection of FCA, AIA rats were randomly divided into NS group, BiP@IOK group and BiPM@IOK group. (1) NS group: 100 μL normal saline; (2) BiP@IOK group: 100 μL BiP@IOK hydrogel; (3) BiPM@IOK group: 100 μL BiPM@IOK hydrogel. The dose of BiNS/PEI in (2) and (3) groups was 400 μg/kg. After 4 h administration, the synovium was irradiated with 1 W/cm^2^ 808 nm laser for 5 min. The temperature was measured by infrared thermal imager. The curve with time as the horizontal coordinate and temperature change as the vertical coordinate was drawn.

**BiPM@IOK Anti-inflammatory effects *in vivo***

The group and treatment were as followed: (1) Control group: healthy rats; (2) NS group: 100 μL normal saline; (3) MTX group: 100 μL MTX (MTX: 200 μg/kg); (4) MTX@IOK group: 100 μL MTX@IOK hydrogel (MTX: 200 μg/kg); (5) BiP@IOK group: 100 μL BiP@IOK hydrogel (BiNS/PEI: 400 μg/kg); (6) BiPM@IOK group: 100 μL BiPM@IOK hydrogel (MTX: 200 μg/kg, BiNS/PEI: 400 μg/kg); (5) and (6) groups were irradiated with 1 W/cm^2^ 808 nm laser for 5 min after 4 h of administration and 0.1 W/cm^2^ 660 nm laser for 20 min after 24 h of administration. The rats were treated once a week. The thickness of the right posterior paws and the left posterior paws were measured every 3 days. The ratio of was calculated as an indicator to evaluate the degree of joint swelling. The rats were killed on the 35th day, that is, 3 weeks after administration. Images of the right feet of the rats were recorded and the degree of joint swelling in each group was compared to evaluate the therapeutic effect. Concentrations of TNF-α and IL-1β in synovium tissue were detected by ELISA kit. The right feet were collected and fixed with 4% paraformaldehyde for 48 h before decalcification. After that, safranine O staining was performed to evaluate cartilage damage and repair.

***In vivo* biosafety studies**

Hematoxylin-eosin staining (H&E) analysis was conducted to evaluate the safety of BiPM@IOK *in vivo*. (180 ± 20) g healthy male SD rats were divided into 2 groups with 3 rats in each group. Rats injected with 100 μL normal saline in joint synovium were used as control group (NS group), and rats injected with 100 μL BiPM@IOK hydrogel were used as treatment group (BiPM@IOK group) (MTX: 200 μg/kg, BiNS/PEI: 400 μg/kg). After three weeks of administration, the rats were killed. The heart, liver, spleen, lung and kidney tissues of the two groups were dissected for H&E staining analysis. Blood was collected in the anticoagulant tube and levels of RBC, WBC, ALT, AST, BUN and CR were analyzed.


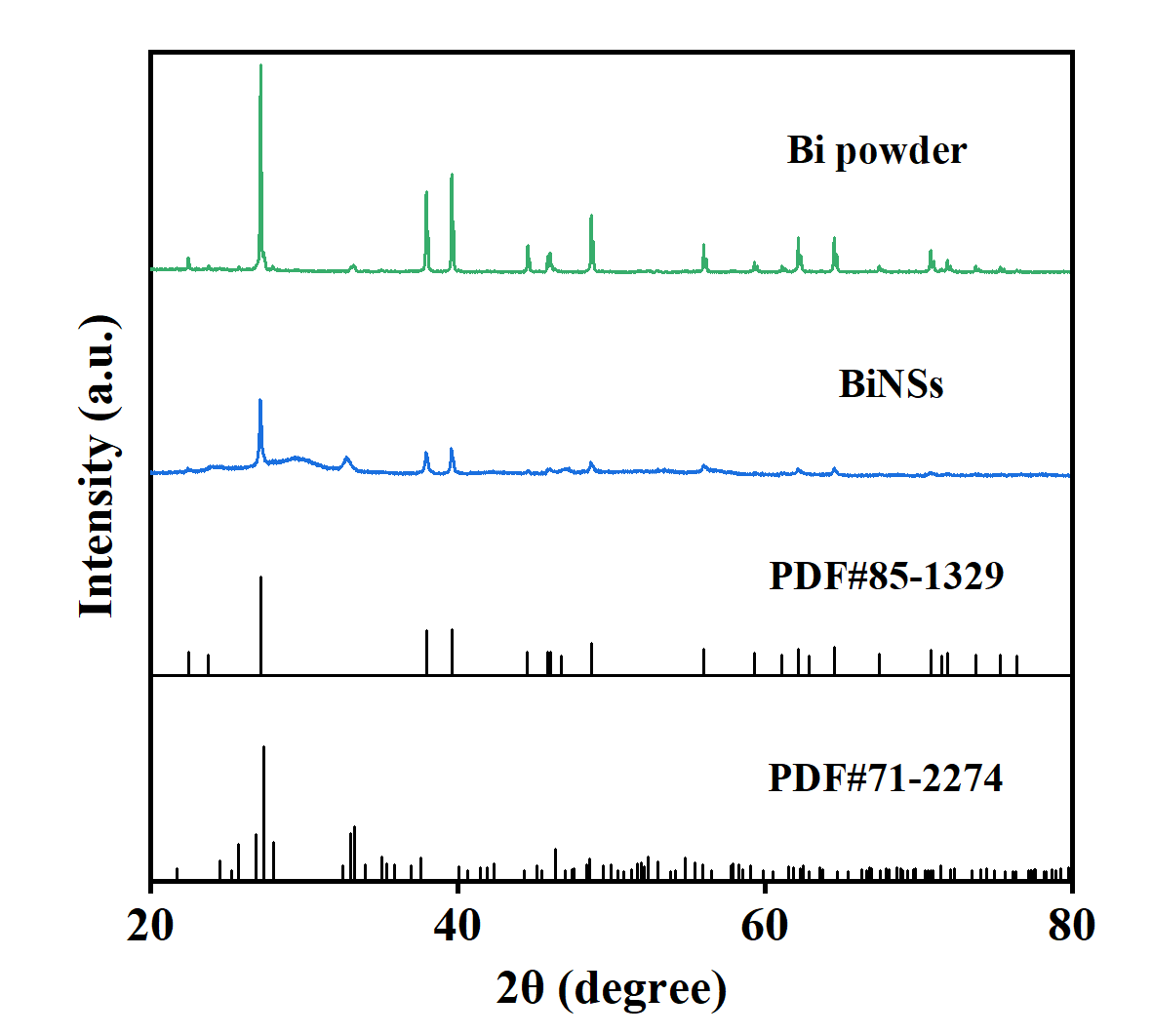


Fig S1. XRD patterns of Bi powder and BiNS and the corresponding standard patterns of Bi (PDF#85-2329) and Bi_2_O_3_ (PDF#71-2274).


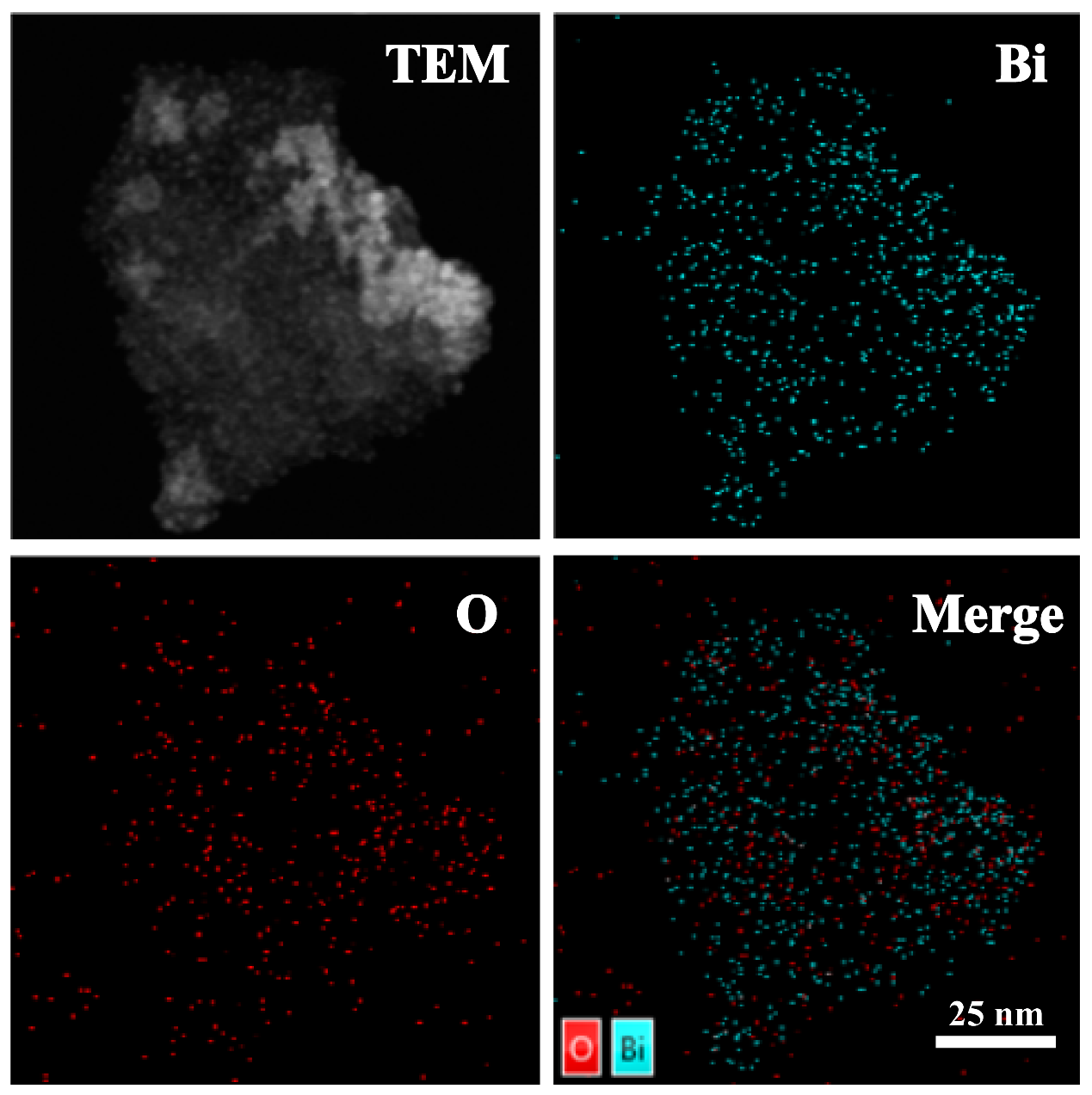


Fig S2. TEM and EDS mapping images of BiNS. Scale bar: 25 nm.


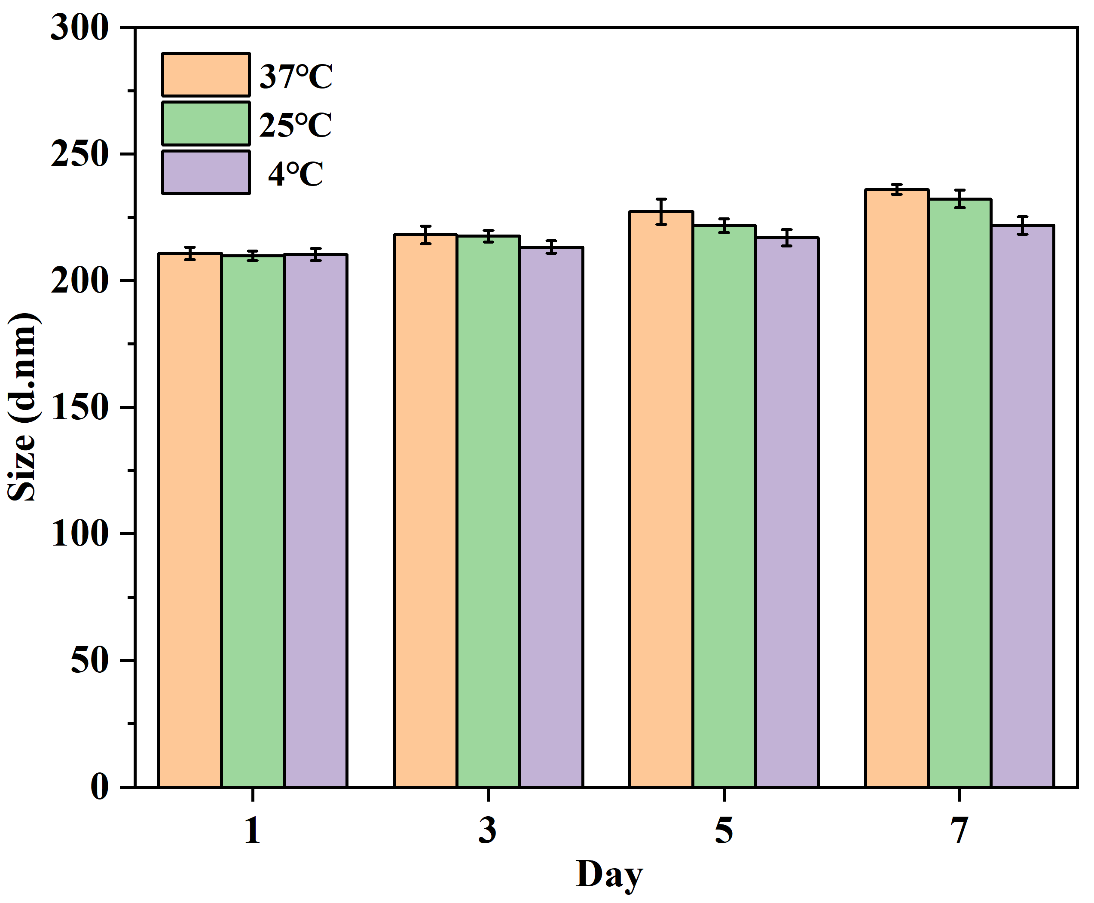


Fig. S3 Particle size of BiNS/PEI within 7 days at 4, 25 and 37℃


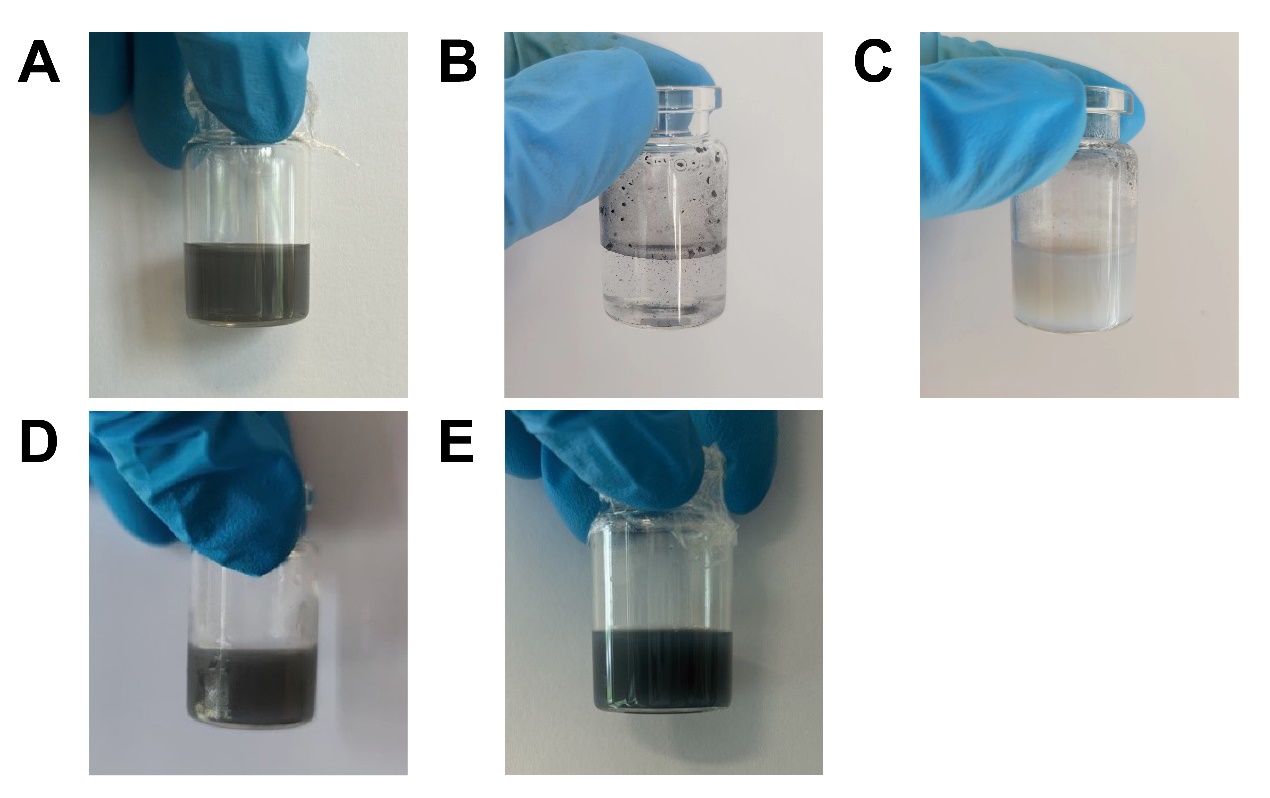


Fig. S4 Images of BiNS suspended in ethanol(A), lyophilized BiNS powder re-suspended in water without (B) or with (C) ultrasonication, BiNS/PEI in water (D), BiNS/PEI stored in water for 3 months (E).


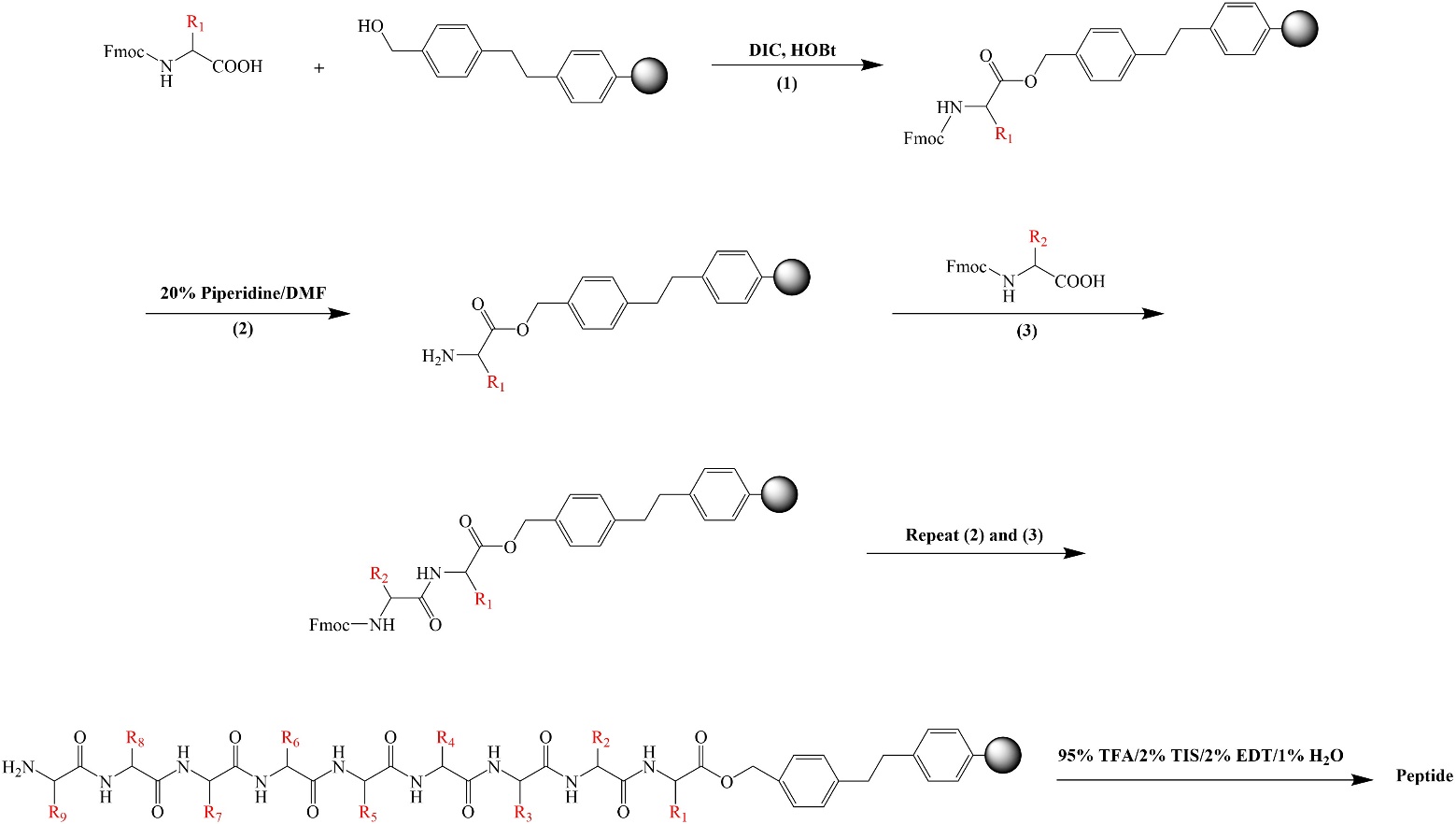


Fig. S5 Synthesis route of designed peptide on Wang resin


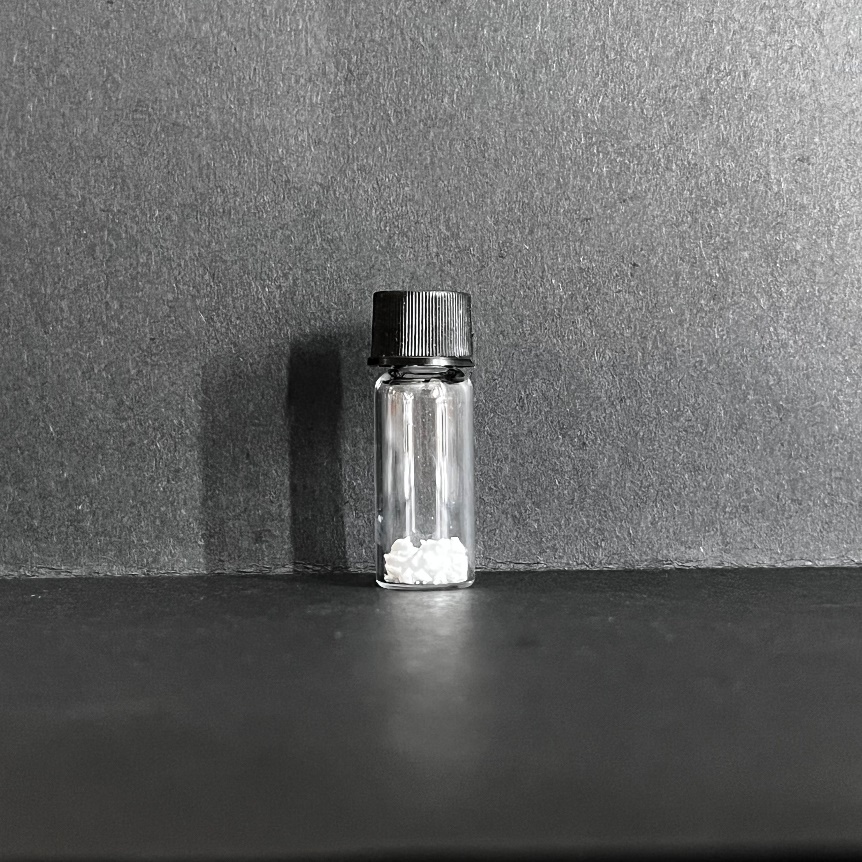


Fig. S6 Morphology of IOK peptide


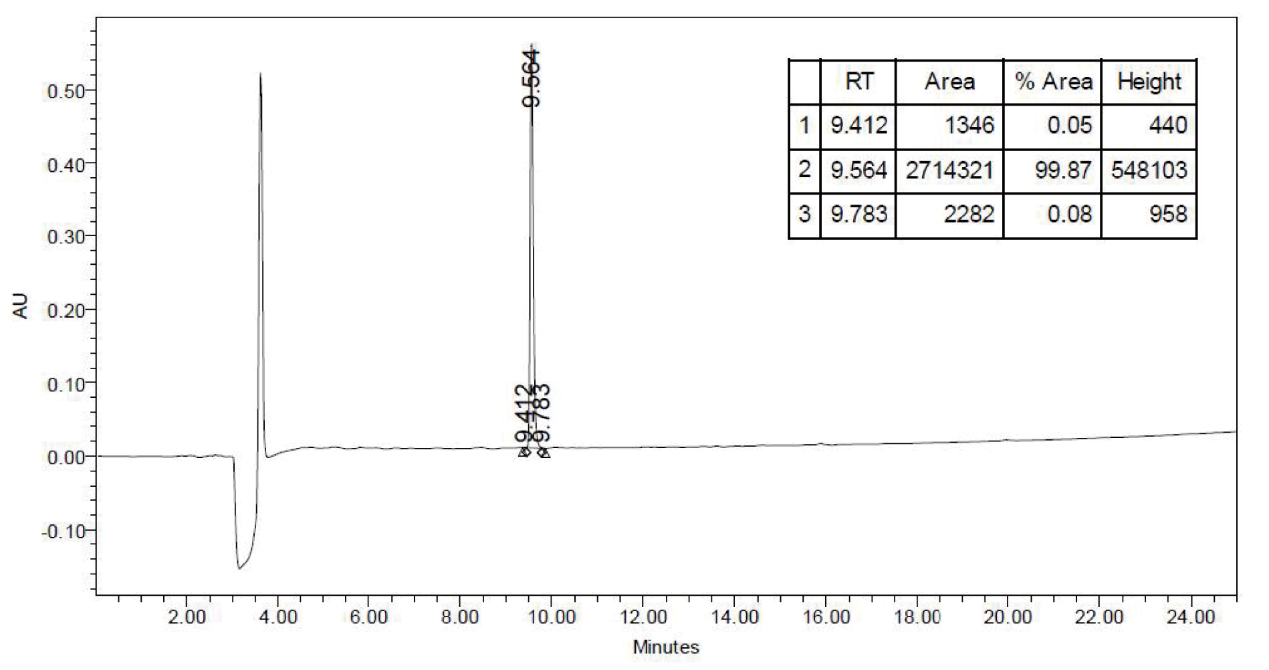


Fig. S7 High performance liquid chromatography of IOK peptide.


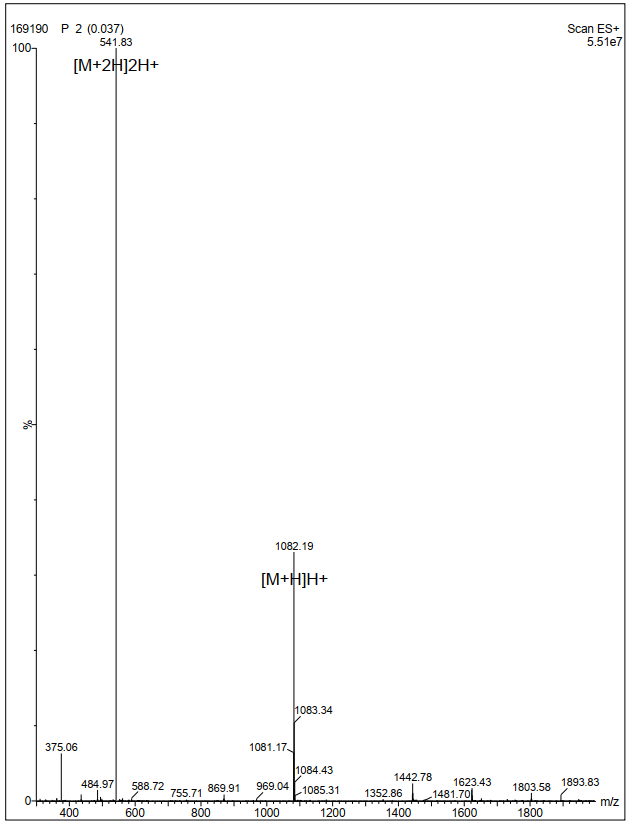


Fig. S8 Mass spectrum of IOK peptide


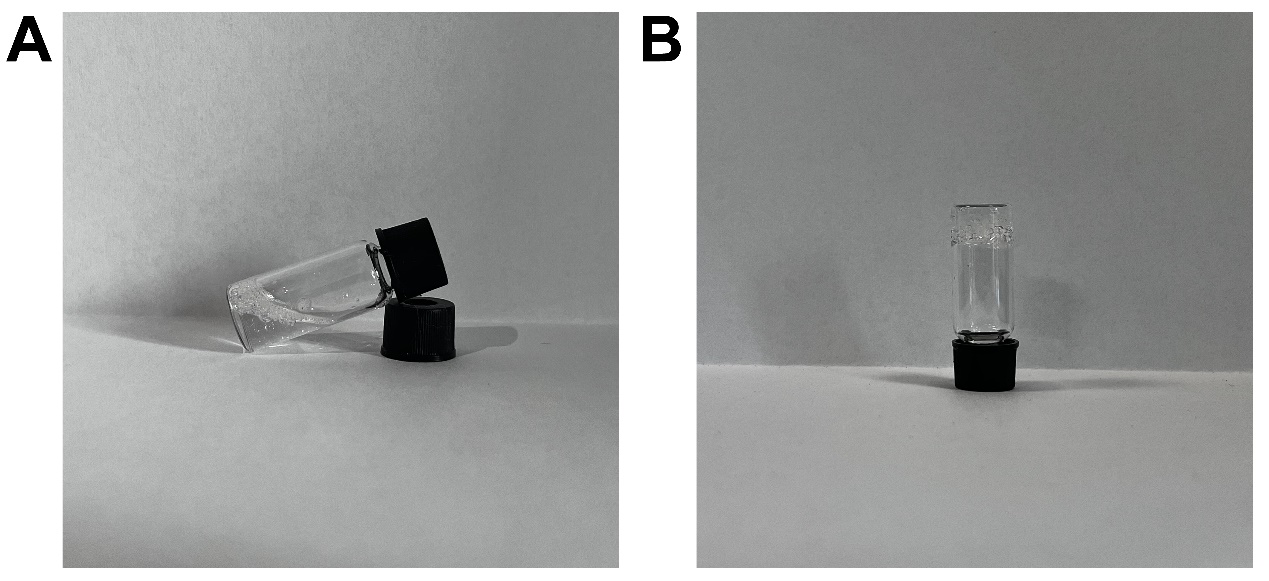


Fig. S9 Morphology of blank IOK hydrogel before (A) and after (B) pH adjusting.


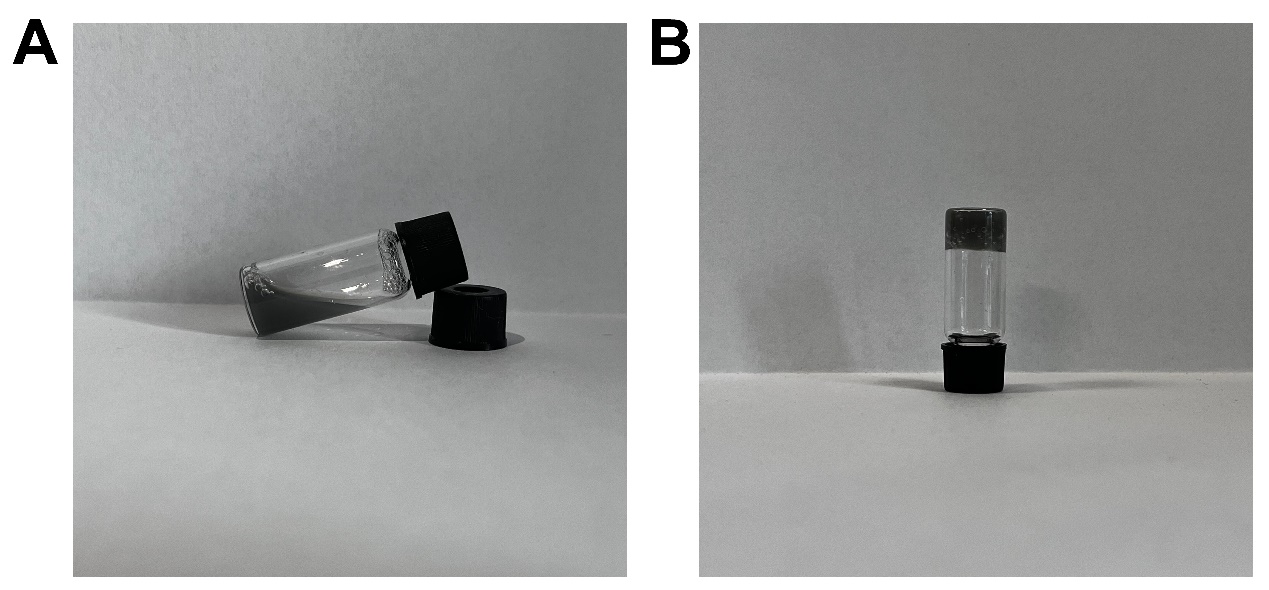


Fig. S10 Morphology of BiPM@IOK hydrogel before (A) and after (B) pH adjusting.


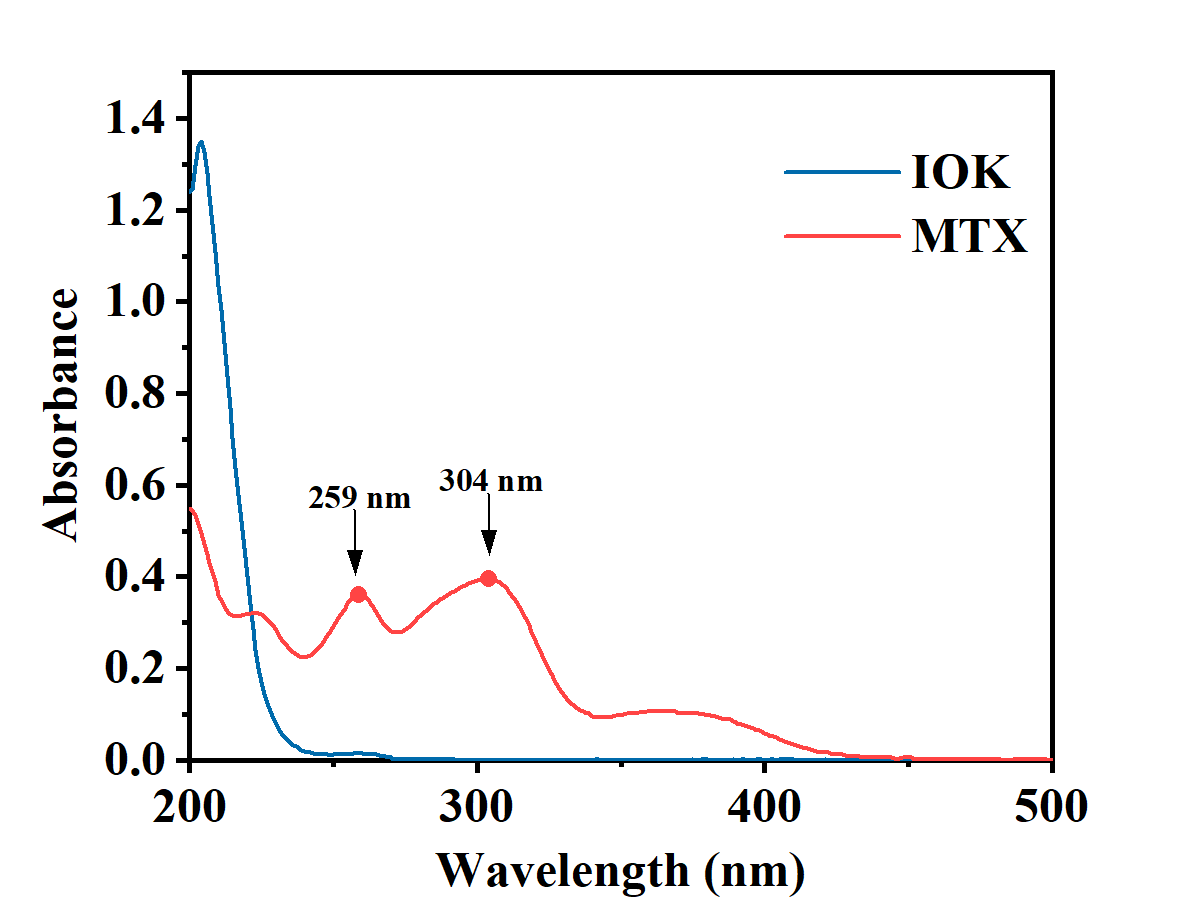


Fig. S11 UV-vis-NIR absorption spectra of IOK peptide and MTX


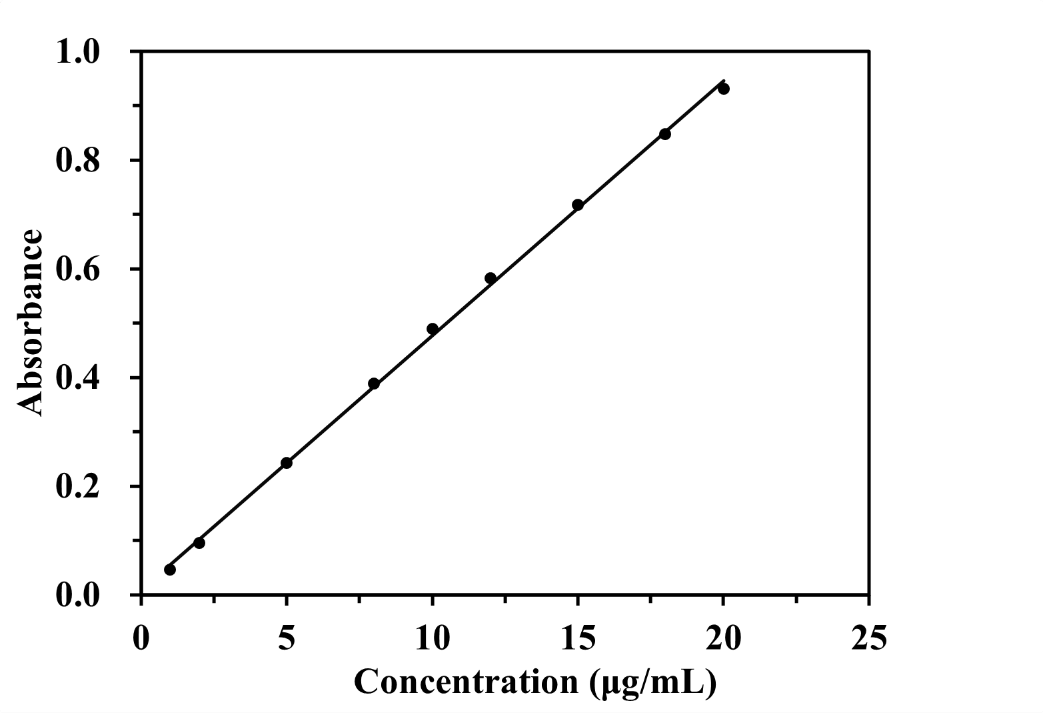


Fig. S12 Standard curve of MTX in PBS solution. A = 0.0469x + 0.0081，R^2^ = 0.9991


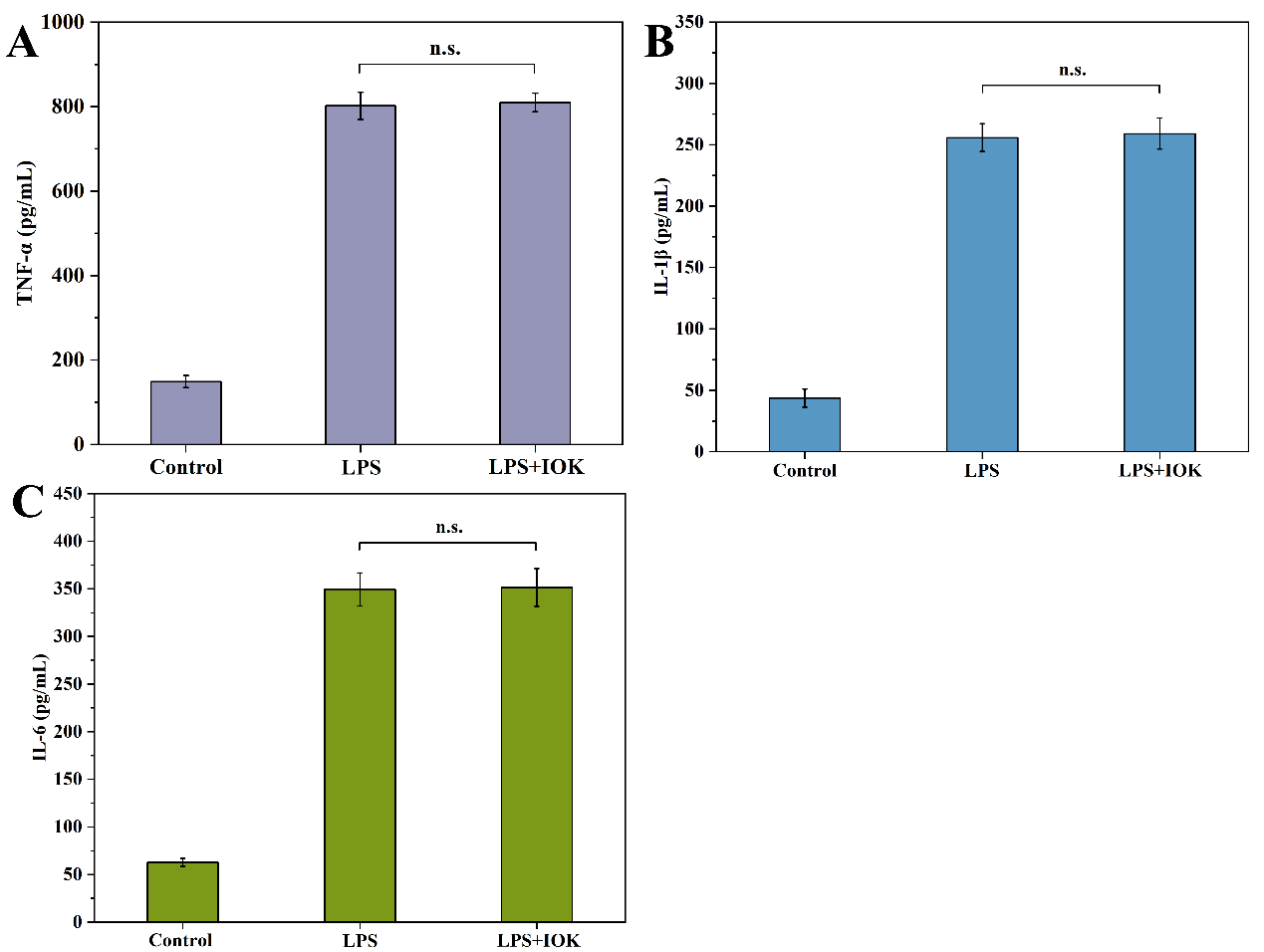


Fig. S13 Concentration of TNF-α (A), IL-1β (B) and IL-6 (C) secreted by RAW 264.7 cells treated with LPS and LPS+IOK. n.s.: no significance.


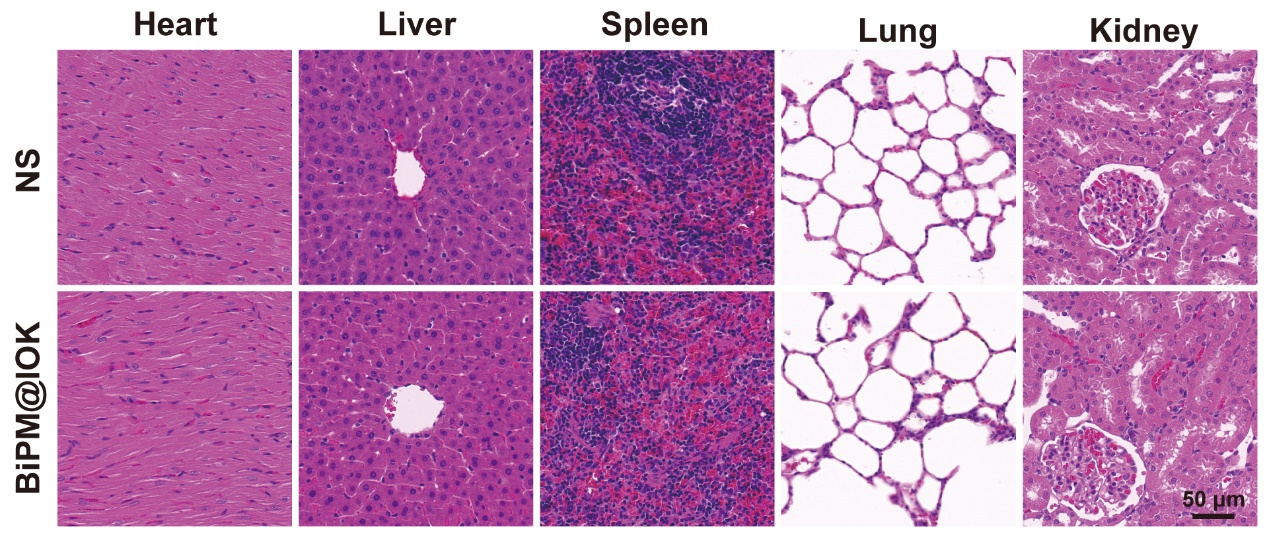


Fig. S14 H&E-staining images of main organs dissected from rats with local synovium injection of NS or BiPM@IOK. Scale bar: 50 μm.


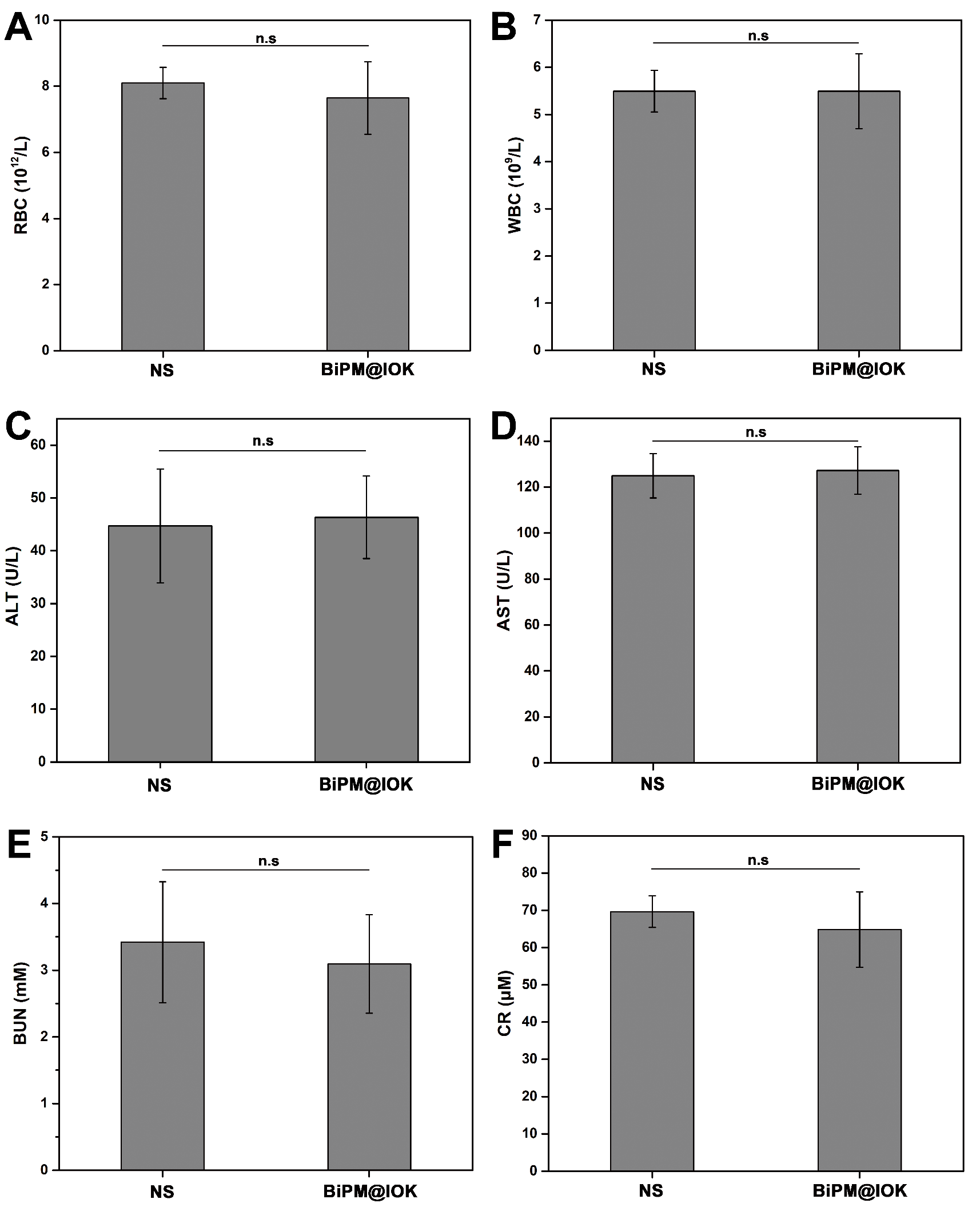


Fig. S15 Hematological parameters (A) RBC, (B)WBC and serum levels of (C) ALT, (D) AST, (E) BUN, (F) CR rats collected from treated with Normal Saline or BiPM@IOK. n=5, n.s: no significance.

Table S1. Chromatography condition of HPLC

| Type | Condition |
| --- | --- |
| Analytial Column type | SHIMADZU Inertsil ODS-SP (4.6×250 mm, 5 μm) |
| Pump A | 0.1% Trifluoroacetic/H_2_O (V/V) |
| Pump B | 0.1% Trifluoroacetic/Trifluoroacetic (V/V) |
| Total flow | 1 mL/min |
| Injection volume | 30 μL |
| Wavelength | 220 nm |

Video. S1 Absorbing, injecting and recovering process of BiP@IOK
